# Caffeine improves mitochondrial dysfunction in the white matter of neonatal rats with hypoxia-ischemia through deacetylation: a proteomic analysis of lysine acetylation

**DOI:** 10.3389/fnmol.2024.1394886

**Published:** 2024-04-30

**Authors:** Yajun Zhang, Yuqian Wang, Haiping Dou, Shanshan Wang, Danyang Qu, Xin Peng, Ning Zou, Liu Yang

**Affiliations:** ^1^Department of Anesthesiology, Dalian Women and Children's Medical Group, Dalian, Liaoning, China; ^2^Department of Pediatrics, The Second Hospital of Dalian Medical University, Dalian, Liaoning, China; ^3^Department of Pediatrics, Shengjing Hospital of China Medical University, Shenyang, Liaoning, China

**Keywords:** white matter damage, caffeine, high performance liquid chromatography, tandem mass spectrometry, lysine acetylation, microtubule associated protein tau

## Abstract

**Aims:**

White matter damage (WMD) is linked to both cerebral palsy and cognitive deficits in infants born prematurely. The focus of this study was to examine how caffeine influences the acetylation of proteins within the neonatal white matter and to evaluate its effectiveness in treating white matter damage caused by hypoxia-ischemia.

**Main methods:**

We employed a method combining affinity enrichment with advanced liquid chromatography and mass spectrometry to profile acetylation in proteins from the white matter of neonatal rats grouped into control (Sham), hypoxic-ischemic (HI), and caffeine-treated (Caffeine) groups.

**Key findings:**

Our findings included 1,999 sites of lysine acetylation across 1,123 proteins, with quantifiable changes noted in 1,342 sites within 689 proteins. Analysis of these patterns identified recurring sequences adjacent to the acetylation sites, notably YKacN, FkacN, and G ^*^^*^^*^ GkacS. Investigation into the biological roles of these proteins through Gene Ontology analysis indicated their involvement in a variety of cellular processes, predominantly within mitochondrial locations. Further analysis indicated that the acetylation of tau (Mapt), a protein associated with microtubules, was elevated in the HI condition; however, caffeine treatment appeared to mitigate this over-modification, thus potentially aiding in reducing oxidative stress, inflammation in the nervous system, and improving mitochondrial health. Caffeine inhibited acetylated Mapt through sirtuin 2 (SITR2), promoted Mapt nuclear translocation, and improved mitochondrial dysfunction, which was subsequently weakened by the SIRT2 inhibitor, AK-7.

**Significance:**

Caffeine-induced changes in lysine acetylation may play a key role in improving mitochondrial dysfunction and inhibiting oxidative stress and neuroinflammation.

## 1 Introduction

White matter damage (WMD), a multifactorial disease resulting from cerebral hypoxia, ischemia, and inflammatory responses to damage in late pregnancy or in the perinatal period (Bano et al., 2017), is a common type of brain injury in premature infants. It is the leading cause of long-term adverse neurological prognoses in premature infants. With the significant increase in the survival rate of critically ill, premature infants, the incidence of WMD and its sequelae has also increased (Molines et al., 2019). WMD often involves limb movement disorders, cognitive behavioral disorders, and intellectual disabilities, as well as visual and auditory disorders, and some infants may experience seizures or even develop epilepsy (Cooper et al., 2017; Younge et al., 2017).

In recent years, some scholars have proposed a third stage of hypoxic-ischemic (HI) nerve injury, which involves neuroinflammation and the continuous release of cytokines and other harmful factors, as well as epigenetic modifications, which may damage axonal growth, synaptogenesis, and neurogenesis (Fleiss et al., 2021). Studying the interaction between epigenetics and neural injury repair could present a novel and significant focus area for the investigation of neonatal brain injury.

Epigenetics involves the transmission of alterations in gene expression or cellular phenotypes that are not attributed to modifications in the DNA sequence but possess the capacity to be inherited by subsequent generations (Ogino et al., 2013). Epigenetic mechanisms have various functions, including in neurogenesis, neuronal plasticity, learning, memory, and have been implicated in disorders like depression, addiction, schizophrenia, and cognitive impairment (Stilling et al., 2014; Landgrave-Gómez et al., 2015). However, our understanding of the specific impact of epigenetics on the nerve damage repair remains limited.

Lysine acetylation is a widespread reversible post-translational modification (PTM) present in a variety of organisms. It is tightly controlled by two distinct sets of enzymes, namely lysine acyltransferases and lysine deacetylases, which have opposing activities (Yang and Seto, 2007). Lysine acetylation is crucial in altering protein functionality and participates in multiple metabolic pathways. The broad spectrum of effects included alterations in how DNA is packaged and read, regulation of cell division, RNA processing, structure and movement within cells, transport across membranes, and key metabolic processes such as sugar breakdown, sugar synthesis, and the citric acid cycle (Choudhary et al., 2009; Zhao et al., 2010). Most of these processes predominantly occur within the mitochondria. In neural cells, lysine acetylation can affect oxidative stress and mitochondrial function through various pathways. Firstly, mitochondrial proteins play a role in the tricarboxylic acid (TCA) cycle through lysine acetylation, thereby affecting ATP production. In addition, mitochondrial ROS is the main product of the electron transport chain. The lysine acetylation state of proteins within the electronic transport chain can also affect their efficiency. Therefore, acetylation of lysine can affect the composition of the electron transport chain, alter its activity, affect ROS levels, and thus affect the overall energy generation and transmission of mitochondria. Therefore, understanding the regulation of lysine acetylation can help develop mitochondrial targeted neuroprotective therapies. Currently, it is known that mitochondrial dysfunction and elevated ROS are associated with a range of neurological disorders, including Alzheimer's disease, Parkinson's disease, and Huntington's dance (Arduíno et al., 2010; Toker et al., 2021; Masaldan et al., 2022; Pradeepkiran et al., 2023). Recent studies have elucidated that oligodendrocytes facilitate an enhancement in axonal energy metabolism by translocating SIRT2, which in turn induces deacetylation of mitochondrial proteins (Chamberlain et al., 2021). Notably, in Parkinson's disease (PD), the deacetylation of succinate dehydrogenase complex, subunit A (SDHA), mediated by SIRT3, activates mitochondrial complex II, suggesting a critical role for the SIRT3-SDHA axis in promoting metabolic efficacy and neuroprotection (Shen et al., 2023). Consequently, strategic modulation of lysine acetylation may offer a therapeutic avenue for reducing oxidative stress, reinstating mitochondrial integrity, and safeguarding neuronal health. Nevertheless, the precise implications of this regulatory mechanism in the context of WMD remain to be delineated. Hence, modulating lysine acetylation to enhance mitochondrial function could potentially serve as a crucial neuroprotective approach in WMD.

Caffeine, a methylxanthine drug, has been utilized in neonatal intensive care units for more than 30 years to manage neonatal apnea. Experimental studies indicate that caffeine has the potential to reduce neural apoptosis in the developing brain and alleviate hypoxia-induced ventricular enlargement and white matter loss (Potter et al., 2018). Recent research suggests that caffeine's neuroprotective effects may be linked to its capacity to mitigate neuroinflammation, oxidative stress, and regulate epigenetic processes (Pereira-Figueiredo et al., 2022; Ding et al., 2023). In our prior proteomic investigations, we have conclusively identified and corroborated SIRT2 as a pivotal modulator for caffeine's neuroprotective outcomes in hypoxic-ischemic WMD (Yang et al., 2022a). Acting as a deacetylase, SIRT2 is instrumental in the orchestration of neuronal mitochondrial functionality (Silva et al., 2017; Chamberlain et al., 2021). Across the spectrum of acute neural insults and progressive neurodegenerative disorders, sirtuins assume a critical role by modulating mitochondrial acetylation. Consequently, we posit that sirtuins represent a viable therapeutic target for a gamut of traumatic and degenerative neural pathologies (Yang et al., 2020). Hence, our current inquiry probes the potential of caffeine to modulate protein acetylation via SIRT2, thereby influencing mitochondrial dynamics in the context of hypoxic-ischemic cerebral injury.

In this study, our primary objective was to assess global alterations in acetylated proteins within the midbrain white matter of neonatal rats using tandem mass tag labeling and Kac affinity enrichment techniques coupled with high performance liquid chromatography-mass spectrometry (HPLC-MS). Additionally, we performed a bioinformatics analysis to explore potential molecular mechanisms underlying caffeine's beneficial effects on brain function in HI WMD.

## 2 Materials and methods

### 2.1 Animals

The Ethics Committee for Animal Research at China Medical University in Shenyang has sanctioned experiments involving animals. We procured neonatal Sprague-Dawley rodents from the Liaoning Changsheng Biotechnology company, situated likewise in Shenyang. These rodents were accommodated in a habitat that maintained a consistent 12-h cycle of light and darkness, with uninterrupted access to food and water.

### 2.2 Model establishment

To induce cerebral WMD in neonatal rats, we followed the procedure described below which was previously employed by our research team (Vannucci et al., 1999; Cheng et al., 2015). At the age of 3 days, both male and female Sprague-Dawley rodents were sedated using isoflurane before being positioned on their backs on the surgery table. Under an anatomical microscope, we exposed the left common carotid artery. The rats were randomly assigned to one of three groups: the Sham group, HI group, and Caffeine group. In the HI group, we permanently ligated both ends of the left common carotid artery using sterile needle sutures and then severed the segment between the ligations. The incision was closed with sutures, and the entire surgery lasted for ~8–10 min. Following recovery from anesthesia, the rats were reunited with their dams for a period of 1 h before being transferred to a low oxygen box maintained at a constant temperature of 37°C. In the low oxygen box, a gas mixture composed of 8% oxygen and 92% nitrogen was continuously administered at a flow rate of 2 L/min for a duration of 2.5 h, resulting in an oxygen concentration of 8%. The Sham group underwent left common carotid artery dissection without ligation or exposure to hypoxia.

From post-natal (P) days 2 to 6, the Caffeine group were administered 20 mg/kg/day caffeine citrate via abdominal cavity injections once daily for five consecutive days. In the Sham and HI group, an equivalent volume of physiological saline was injected instead. The caffeine citrate used in the study was sourced from Casey Pharmaceuticals (Casey Pharmaceuticals, Parma, Italy). Furthermore, in the Caffeine+AK-7 group, rats were subjected to treatment with both caffeine and the sirtuin 2 (SIRT2) inhibitor 3-(1-azepanylsulfonyl)-N-(3-bromphenyl) benzamide (AK-7) (s591401, Selleck Chemicals, USA). AK-7 was administered intraperitoneally at a dosage of 20 mg/kg/day for a period of 5 days, commencing from day 2 to day 6 after birth.

### 2.3 Sample collection and preparation

Two weeks after the model was established (on the 17th postnatal day), we harvested and immediately preserved the brain's white matter by freezing it on a precooled platform using liquid nitrogen. For preservation, we stored the brain samples at −80°C until further analysis. Each sample was gently thawed, subjected to ultrasonic disruption for finer separation, and then centrifuged at 12,000 revolutions per minute for 10 min at a temperature of 4 degrees Celsius. After centrifugation, we carefully extracted the supernatant from the cellular waste and placed it into a new tube. To deplete the most common 14 proteins, we used a depletion kit from Thermo Fisher Scientific's Pierce™ brand. The protein levels were then measured with a bicinchoninic acid assay kit from Beyotime. For protein digestion, we initially reduced the proteins with 5 mM dithiothreitol at 56 degrees Celsius for half an hour. The alkylation step followed by adding 11 mM iodoacetamide and incubating the solution for 15 min at ambient temperature shielded from light. We diluted the urea concentration to under 2 M using 100 mM tetraethylammonium bromide before adding trypsin at a protein-to-trypsin mass ratio of 1:50, allowing digestion to occur overnight. A secondary digestion was performed for an additional 4 h at a ratio of 1:100. In order to avoid interference in mass spectrometry analysis, we try to avoid the introduction of surfactants during protein extraction and pretreatment of proteome samples. At the same time, before mass spectrometry, we remove non peptide components from the samples through C18 solid-phase extraction to minimize matrix effects.

### 2.4 LC-MS/MS

Tryptic peptides were dissolved in solvent A, which consisted of 0.1% formic acid and 2% acetonitrile in water. The peptide solution was then loaded directly onto a homemade reversed-phase analytical column with dimensions of 25 cm length and 75 μm i.d. The separation of peptides was achieved using solvent B, a gradient ranging from 5% to 25% consisting of 0.1% formic acid in 90% acetonitrile, over a period of 60 min. Subsequently, the gradient was adjusted to range from 25% to 35% over 22 min, followed by a sharp increase to 80% over 4 min. The system was maintained at 80% for the final 4 min. These separations were performed at a constant flow rate of 450 nL/min on an EASY-nLC 1200 UPLC system manufactured by Thermo Fisher Scientific.

The separated peptides were analyzed using a Q ExactiveTM HF-X mass spectrometer manufactured by Thermo Fisher Scientific. The analysis was performed using a nano-electrospray ion source, with an applied electrospray voltage of 2.0 kV. Full MS scans were conducted at a resolution of 60,000, covering a scan range of 350–1,600 m/z. For MS/MS analysis, up to 20 of the most abundant precursor ions were selected, with a dynamic exclusion time of 30 s. HCD fragmentation was utilized, with a normalized collision energy of 28%, and the resulting fragments were detected using an Orbitrap at a resolution of 30,000. The first fixed mass was set to 100 m/z. The automatic gain control target was set to 1E5 with an intensity threshold of 3.3E4, and the maximum injection time was set to 60 ms.

### 2.5 Database search

MaxQuant software (v.1.6.15.0) was utilized to examine the gathered tandem mass spectrometry data. By employing the SwissProt human protein database, which consists of 20,422 protein records, we successfully matched peptides. A reversed decoy database was integrated to evaluate the rate of incorrect identifications. The enzyme trypsin/P was assumed to have specificity, with provisions for potential missed cleavages capped at two. The initial search allowed for a mass deviation of 20 parts per million (ppm) for precursor ions, which was later narrowed to 5 ppm. A quality deviation of 20 ppm basically covers the detection of the vast majority of peptide segments while maintaining the accuracy of the results. Due to the independence of quality deviation from peptide abundance, quality accuracy has no effect on the results of low abundance peptide segments. A mass variance of 0.02 Dalton was permitted for fragment ions.

### 2.6 Bioinformatic analysis

Criteria set to pinpoint acetylated proteins with altered expression included a *p*-value under 0.05 and a fold change above 1.2 or below 0.83. To characterize conserved sequences around acetylation sites, a 21-residue span (10 residues on either side of the acetylation site) was examined using the motif discovery application Soft motif-x (Chou and Schwartz, 2011). This application is adept at detecting recurring motifs within protein sequences. The secondary structure of the proteins was predicted using NetSurfP, with protein sequences from the database providing the background for predictions and *t*-tests being conducted for statistical evaluations (Klausen et al., 2019). Subcellular localization predictions were made with WoLF PSORT, using default settings and the organism-specific background (Horton et al., 2007). We analyzed the functional pathways and gene ontology of the altered proteins using OmicsBean, applying hypergeometric tests for enrichment with a significance cut-off of *p* < 0.05. The functions disrupted by these protein changes were explored using the Ingenuity Pathway Analysis tool, with significance determined by Fisher's exact test (*p* < 0.05) and p-scores calculated accordingly (Krämer et al., 2014). The STRING database (v9.1) was employed for analyzing protein interaction networks and the functional domains of acetylated lysines. Fuzzy c-means clustering was used to elucidate gene clusters while GO, KEGG, and protein domain analyses provided insight into the biological processes for each protein cluster, considering terms with *p* < 0.05 as significant.

### 2.7 Electron microscopy

A fortnight subsequent to the creation of the animal model, a sample of six rodents from each group was euthanized in a manner that minimized suffering. The brains were then extracted, and the cerebral slices from the left side were precisely cut into cubic millimeter sections, ranging from 1.5 mm anterior to 0.5 mm posterior to the bregma landmark. These samples were preserved in a fixative composed of 4% paraformaldehyde and 2.5% glutaraldehyde. Further processing of the samples included treatment with 1% osmium tetroxide, a stepwise dehydration in an acetone series, and finally, embedding in an epoxy resin for subsequent slicing. Sections of extreme thinness were obtained using an ultramicrotome, then stained with uranyl acetate and lead citrate, and examined under a JEM-1400 transmission electron microscope manufactured by JEOL, located in Tokyo, Japan.

### 2.8 Western blotting

Fourteen days post-model creation, the rats were humanely terminated. The cortex was removed, and brain samples were extracted from the ligation-adjacent periventricular zones, immediately chilled, and preserved at −80°C. Samples were lysed in RIPA buffer, supplemented with phosphatase inhibitors from Sigma-Aldrich, France. Nuclear proteins were sourced utilizing a Solarbio kit, and protein levels quantified via a BCA assay from Sigma-Aldrich. Proteins (50 μg each) underwent SDS-PAGE (4–20%) and were then applied to PVDF sheets. After blocking with 5% skim milk for 2 h, the membranes were incubated at 4°C overnight with a selection of primary antibodies from Abcam and Proteintech, followed by corresponding secondary antibodies from Proteintech, and visualized using ECL substrates from Thermo Fisher Scientific. Band intensities were quantified using ImageJ and normalized to β-tubulin, β-actin, or Lamin B markers.

### 2.9 Immunoprecipitation and immunoblotting

The isolated tissue was resuspended in RIPA buffer in the presence of protease inhibitors. For immunoprecipitation assays, a Pierce Co-IP Kit was employed. Lysates were precleared with control Ig and protein A/G beads at 4°C for 1 h. Post-centrifugation, supernatants were collected; a portion was reserved for Western blot analysis, while the remainder was incubated with Tau antibodies overnight at 4°C. Protein A/G beads were introduced for a subsequent 4 h incubation. The precipitates were centrifuged, washed with PBS, eluted, and analyzed via Western blot for acetyl-lysine.

### 2.10 Real-time polymerase chain reaction (PCR)

The assessment of Mapt gene expression in the tied-off section of the paraventricular brain area was performed using RT-PCR. To prepare RNA samples, the left segment of the midbrain within this region was pulverized, homogenized, and then spun down at a temperature of 4°C (14,000 rpm for 15 minutes). The process of extracting the total RNA was executed with the aid of a TRIzol reagent from Takara Bio, located in Dalian, China. Subsequently, the conversion of RNA to cDNA was accomplished using the HiScript QRT SuperMix specifically designed for qPCR, which includes a gDNA removal feature (product R123-01 from Vazyme Biotech Co., Ltd., Nanjing, China). The RT-PCR analysis was conducted with the ChamQ universal SYBR qPCR master mix (product code Q711 from Vazyme Biotech Co., Ltd.), and the Mapt gene's relative expression was quantified by employing the 2^−ΔΔ^CT technique, utilizing the Gapdh gene as a normalization control. Primer sequences for this analysis were custom-developed by the Shanghai Biotechnology Service Co., Shanghai, China.Mapt forward 5′-ACACATCTCCACGGCACCTCAG-3′ and reverse 5′-GCGGACACTTCATCGGCTAACG-3′ and Gapdh forward 5′-GATGGTGAAGGTCGGTGTGA-3′ and reverse 5′-TGAACTTGCCGTGGGTAGAG-3′.

### 2.11 Mitochondrial membrane potential (MMP) assay

To investigate the effect of caffeine on MMP in brain white matter tissue cells after damage, cells were collected by brain tissue grinding, centrifugation, and resuspended in staining buffer containing JC-1 fluorescent probe (C2006, Beyotime, China). After 20 min of dark staining at 37 °C, the proportion of red fluorescence to green fluorescence in brain tissue cells was detected and analyzed using FCM to reflect the changes of MMP in brain tissue cells.

### 2.12 Statistical analyses

Data were expressed as mean ± SEM. Group differences were determined by one-way ANOVA, using GraphPad Prism, with a significance threshold set at a *p* < 0.05.

## 3 Results

### 3.1 Proteome-wide identification and analysis of lysine acetylation sites following caffeine administration in neonatal rats with HI-induced WMD

**Figure 1:**
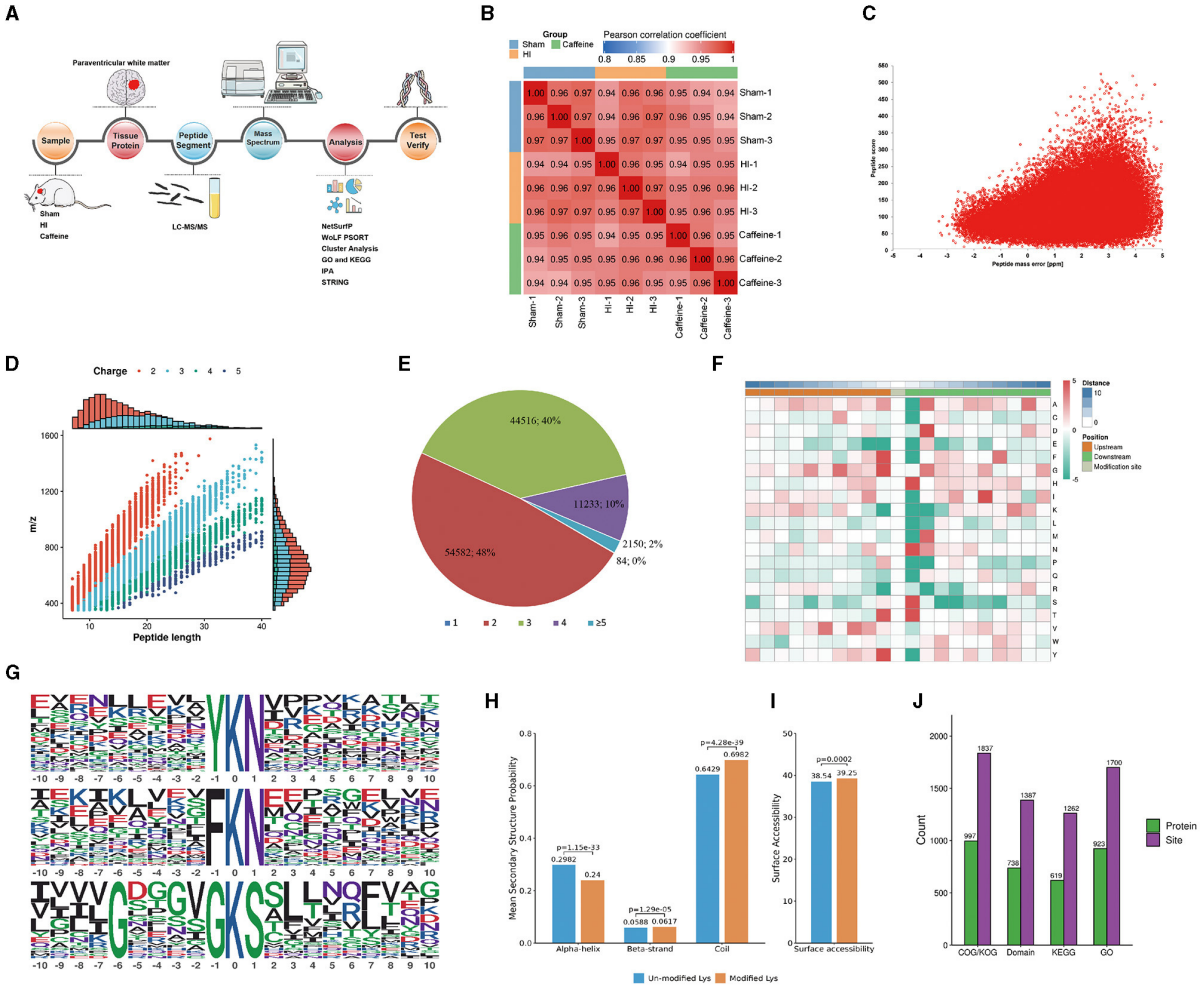
Proteome-wide identification and analysis of lysine acetylation sites in neonatal rats with hypoxia-ischemia-induced white matter disease administered caffeine. **(A)** Flow chart of proteome-wide identification and analysis of lysine acetylation sites. **(B)** Reproducibility analysis of three repeated trials using Pearson's correlation coefficient. **(C)** Mass error distribution of all of the identified peptides. **(D)** Peptide length distribution. **(E)** Pie chart illustrating the number of lysine acetylation sites per protein. **(F)** Heat map of the amino acid compositions of the acetylation sites. **(G)** Acetylation motifs and the conservation of acetylation sites. **(H)** Probabilities of lysine acetylation in different protein secondary structures. **(I)** Predicted surface accessibility of acetylated sites. **(J)** Comprehensive functional annotation of the identified proteins. Sham, Sham group; HI, HI group; Caffeine, Caffeine group.

To investigate the impact of caffeine on acetyl group dynamics in the white matter of HI neonatal rats, we utilized an extensive methodology including Sample preparation, tissue protein extraction, Kac antibody affinity enrichment, LC-MS/MS analysis, mass spectrometry, differential analysis, and subsequent identification and validation processes (Figure 1A). The consistency of the experimental data was confirmed through a repeatability analysis of the three replicates (Figure 1B). A total of 1999 lysine acetylation sites were identified across 1,123 proteins, with 1,342 sites quantified in 689 proteins. The quality errors of the identified peptides were assessed, revealing an error distribution centered around zero, with the majority of errors below 5 ppm, demonstrating high accuracy and compliance to the requirements of MS data (Figure 1C). The length distribution of the peptide segments ranged from 7 to 20 amino acids, consistent with enzymatic hydrolysis and MS fragmentation patterns (Figure 1D), indicating appropriate sample preparation. These proteins displayed varying numbers of acetylation sites (Figure 1E). Among the acetylated proteins, 48% had two modification sites, 40% had three sites, and 20% had four sites. Notably, only 2% of the acetylated proteins had one or five acetylation sites.

To analyze the sequence surrounding the acetylation site in rats, we compared the frequencies of 10 amino acids upstream and downstream of the acetylation site (Figure 1F). Downstream of the acetylated lysine, there was an enrichment of aspartic acid (D), histidine (H), methionine (M), asparagine (N), and serine (S), while upstream of the modification site, phenylalanine (F), tyrosine (Y), and valine (V) were enriched. Both the upstream and downstream regions of the modification site showed enrichment of alanine (A), glycine (G), and threonine (T). Notably, acidic amino acids appeared in these motifs, such as G at +5, +3, and +1, and valine (V) at +5 and +3. The acetylated lysine modification site exhibited F, G, T, and Y at position 1; H, N, S, and T at position 1; or A, D, G, M, and N at position 2. These characteristic sequences are likely to represent motifs of acetylated modified peptides. We identified three significantly enriched acetylation site motifs from the quantified lysine acetylation sites, namely YKacN, FKacN, and G^***^GKacS, where Kac represents acetylated lysine and ^*^ represents random amino acid residues. These motifs may indicate specific protein-protein interaction domains, where amino acids (such as Y, F, N) near acetylated lysine (Kac) may have a significant impact on the specificity or affinity of the interaction. Y. F is usually a hydrophobic amino acid that may contribute to hydrophobic interactions within or between proteins. The regulation of protein function by these specific motifs and their acetylation modifications is multifaceted. They regulate various biological processes within cells by affecting protein activity, stability, localization, interactions, and participating signaling pathways (Huntley and Golding, 2002; Iakoucheva et al., 2004). The specific role and importance of these mechanisms depend on the type of protein, cell type, and biological context. These findings highlight the potential functional importance of the amino acids surrounding lysine during acetylation (Figure 1G). To understand the relationship between lysine acetylation and the local secondary structures of acetylated proteins, we conducted secondary structure predictions.

A significant proportion of the acetylation sites were observed within the coil configurations of proteins (69.82%), with less in the α-helix (24.0%) and even fewer in the β-strand (6.17%) regions (Figure 1H). Surface analysis indicated that around 39.24% of these sites were on the exterior of the proteins (Figure 1I). Such a distribution hints at a proclivity for acetylation within the coiled regions of rat proteins.

We conducted a thorough functional annotation of the identified proteins to gain a comprehensive understanding of their functional characteristics (Figure 1J). This annotation involved several aspects including GO analysis, Protein domain analysis, KEGG pathway analysis, COG/KOG functional classification, subcellular localization analysis. Our findings demonstrated that protein acetylation has a significant involvement in various physiological processes.

### 3.2 Characterization of the lysine acetylome in neonatal rats with HI-induced WMD administered caffeine

**Figure 2:**
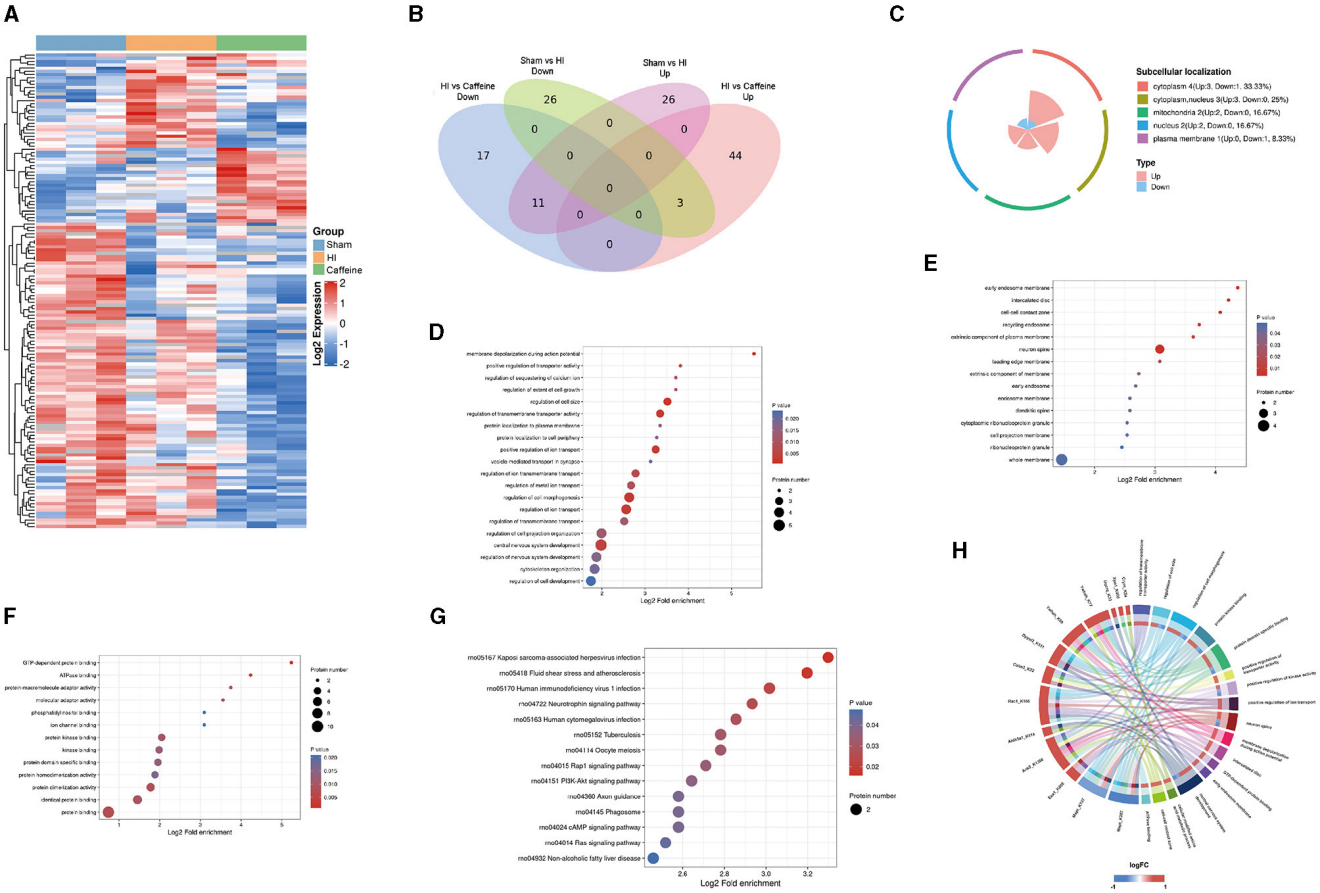
Quantification and identification of proteins with differential expression in the lysine acetylome and bioinformatics analysis in neonatal rats with hypoxia-ischemia (HI)-induced white matter damage administered caffeine. **(A)** Heatmap illustrating protein abundance values of lysine acetylation sites derived from the brains of rats in the Sham, HI, and Caffeine groups (three rats per group). **(B)** Venn diagram depicting differentially expressed lysine acetylation site proteins. **(C)** Subcellular localization of differential lysine acetylome. **(D)** Gene Ontology (GO) of the biological process (BP) of differential lysine acetylome. **(E)** GO of cell composition (CC) of differential lysine acetylome. **(F)** GO of molecular functions (MF) of differential lysine acetylome. **(G)** Kyoto Encyclopedia of Genes and Genomes (KEGG) analysis of differential lysine acetylome. **(H)** GO and KEGG analysis of differentially expressed lysine acetylation site proteins. Sham, Sham group; HI, HI group; Caffeine, Caffeine group.

In exploring the influence of caffeine on acetylation within the white matter of neonatal rats affected by hypoxia-ischemia (HI), we conducted a comparative analysis across three groups, using a significance threshold of *p* < 0.05 and a minimum 1.2-fold change. This led to the identification of 113 acetylation sites across 96 proteins, with specific sites showing increased or decreased acetylation in the HI group. Compared with the Sham and Caffeine groups, 3 acetylation sites in 2 proteins were upregulated in the HI group, and 11 acetylation sites in 10 proteins were downregulated in the HI group (Figures 2A, B). We have described the specific roles of these upregulated and downregulated proteins in neurological diseases (Table 1).

**Table 1:** Differential upregulated and downregulated proteins in Sham group and Caffeine group compared with the HI group.

**Protein description**	**Gene name**	**Position**	**Regulated type**	**The role in nervous system diseases**
Early endosome antigen 1	Eea1	898K	Up	Eea1 regulates the recovery of neurotransmitter receptors during the endocytosis of synapses. Restore the steady-state synaptic plasticity of hippocampal neurons.
Ankyrin 2	Ank2	1306K	Up	ANK2 plays a role in neuronal morphogenesis, including synaptic formation, axonal development, and neural connectivity.
Succinate-semialdehyde dehydrogenase	Aldh5a1	114K	Up	Aldh5a1 is a mitochondrial enzyme involved in the metabolism of neurotransmitters. It is crucial to maintain the normal function of the nervous system.
Ras-related C3 botulinum toxin substrate 1	Rac1	166K	Up	Rac1 is associated with axonal growth and orientation, synaptic formation and plasticity, cell migration, neuroprotective effects, and more. And it can affect mitochondrial function by regulating intracellular ROS production.
Calmodulin-3	Calm3	22K	Up	Calm3, like other members of the calmodulin family, plays an important role in the nervous system. The signaling pathway mediated by calmodulin is closely related to processes such as learning and memory formation, synaptic plasticity, and neuronal survival.
Dihydropyrimidinase-related protein 2	Dpysl2	511K	Up	Dpysl2 plays a crucial role in neuronal differentiation and axonal guidance. This includes regulating microtubule dynamics, neuronal polarity, and axonal guidance.
14-3-3 protein eta	Ywhah	69K, 77K	Up	Ywhah is a protein encoding 14-3-3 η Genes of subtypes (14-3-3 eta). Plays an important role in the development and function of the nervous system, and exhibits significant changes in pathological processes in cardiovascular and cerebrovascular diseases, tumors, metabolic disorders, and specific neurological diseases.
Cytochrome b-c1 complex subunit 8	Uqcrq	33K	Up	Uqcrq is a structural subunit encoded by nuclear DNA of mitochondrial respiratory chain complex III (CIII). Participating in establishing proton gradients on the inner membrane is necessary for ATP synthesis. The Uqcrq gene is associated with certain severe neurodegenerative diseases.
Exportin-1	Xpo1	455K	Up	Xpo1 is associated with its regulatory role in early embryonic development and may affect neuronal development.
Ketimine reductase mu-crystallin	Crym	54K	Up	Crym is associated with Alzheimer's disease, schizophrenia, and bipolar disorder.
Reticulon	Rtn3	102K	Down	Rtn3 is associated with the pathological processes of Alzheimer's disease (AD), regulating nerve cell function and biological processes associated with certain neurodegenerative diseases.
Microtubule-associated protein	Mapt	107K, 207K	Down	Mapt is a gene encoding the microtubule associated protein tau (tau protein). The main function is to promote and stabilize the assembly of microtubules, which are crucial for maintaining neuronal structural stability and supporting nutrient transport to the distal end of the axon. Closely related to neurodegenerative diseases.

Protein description and Protein accession are listed according to the SWISS-PROT database; Up/Down means Sham and Caffeine group VS. HI group.

To investigate the biological functions and associated networks of the 12 proteins with differential expressions in lysine acetylation, we conducted subcellular localization analysis. Initially, we analyzed the subcellular localization patterns of these differentially expressed acetylated proteins. The differential expression of acetylated subcellular localization was mainly observed in the cytoplasm (4, 33.33%), cytoplasmic nucleus (3, 25%), and mitochondria (2, 16.7%). This suggests that the neuroprotective effect of caffeine is primarily achieved through the alteration of acetylation in proteins located in the cytoplasm, cytoplasmic nucleus, and mitochondria (Figure 2C).

Our examination of protein functions affected by acetylation changes highlighted their involvement in a variety of biological activities, from cell size regulation to the development of the central nervous system. These activities are particularly essential during the generation of action potentials (Figure 2D). Furthermore, the enriched cellular components included the primary endosome membrane, neuronal axons, intervertebral discs, and cell junction regions (Figure 2E). Molecular functions with significant enrichment in the analysis included GTP-dependent protein binding, ATPase binding, protein binding, and other molecular functions (Figure 2F).

By delving into the KEGG pathways through functional enrichment analysis, we pinpointed considerable pathway involvement, including neurotrophin and PI3K-Akt signaling (Figure 2G). This analysis corroborated the acetylation patterns we had initially uncovered (Figure 2H).

In summary, these findings suggest that protein acetylation may play a crucial role in various cellular processes occurring in the cytoplasm, nucleus, and mitochondria. Many of the important classical pathways identified are closely associated with energy metabolism and production, suggesting their significance, and warranting further research.

### 3.3 IPA, cluster, and protein-protein interaction networks analyses of differentially-expressed acetylated proteins

**Figure 3:**
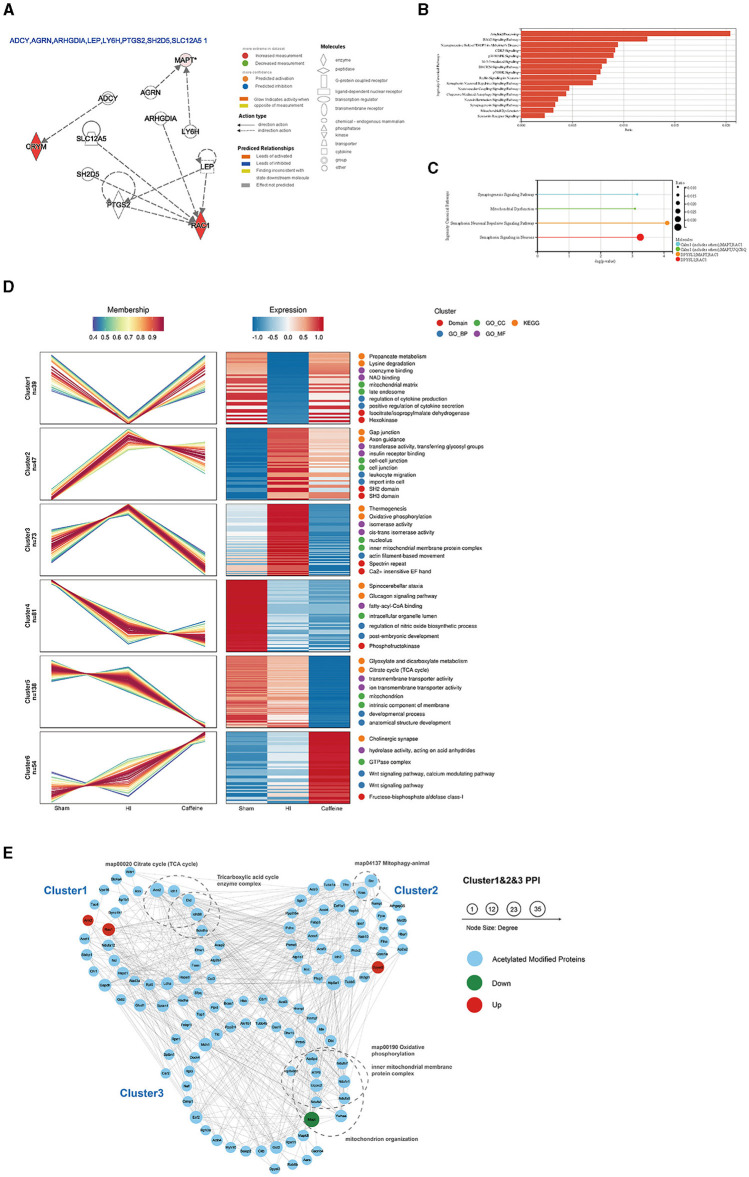
Functional and pathway analyses of the differentially expressed acetylated proteins using Ingenuity Pathway Analysis (IPA), cluster analysis, and protein-protein interaction networks (PPI) analysis. **(A)** Regulatory network in the IPA. **(B)** Disease functions among the downregulated genes. **(C)** Top related canonical pathways in IPA. The different colors in the figure represent different molecular combinations. **(D)** Cluster analysis of the lysine acetylome. The line chart on the left side represents protein expression, and the right side represents expression calorimetry. Each cluster corresponds to a line chart and a heat map. **(E)** Analysis of the PPI networks of Cluster 1, Cluster 2, and Cluster 3. The nodes represent proteins, and the colors of the nodes represent the upregulation or downregulation of these proteins; blue represents acetylated modified proteins in Cluster analysis; red represents upregulated proteins, while green represents downregulated proteins. The size of each node represents the fold-change value of the protein.

Further analysis of the differentially acetylated proteins, which consisted of 10 upregulated proteins and two downregulated proteins, was performed using the IPA database. The analysis revealed a significant enrichment of these proteins in diseases of the nervous system. To gain a better understanding of these differentially expressed proteins, three types of analysis were conducted, namely regulatory network (Figure 3A), disease function (Figure 3B), and classical pathway (Figure 3C) analyses. In the regulatory network analysis, it was found that Mapt was associated with AGRN and LY6H, while RAC1 and CRYM formed a regulatory network with ADCY, ARHGDIA, LEP, PTGS2, SH2D5, and SLC12A5 (Figure 3A). The classical IPA pathway and disease functional pathway were enriched within the 12 differentially expressed proteins. Among the downregulated genes, significant changes related to nervous system diseases such as microglial transformation and neurite neurodegeneration were observed. Additionally, diseases related to mitochondrial transport and degeneration were also noted (Figure 3B).

Moreover, statistically significant pathways identified in the analysis included the synaptogenesis signaling pathway and mitochondrial dysfunction and semaphorin neuronal recursive signaling pathway. Both of which are related to Mapt (Figure 3C). These enriched pathways observed in the IPA analysis are consistent with the results from the KEGG pathway analysis. Many of these pathways are crucial and closely associated with mitochondria. It is worth noting that the downregulated proteins primarily participate in these pathways, suggesting their potential significance and highlighting the need for further investigation.

To investigate the impact of caffeine on the acetylation of lysine in the white matter of HI neonatal rats, we performed an expression pattern clustering analysis on 1999 lysine acetylation sites derived from three groups, namely the Sham, HI, and Caffeine groups. We focused on proteins that exhibited significant changes in the abundance of acetylation modifications across these groups. First, we transformed the relative expression levels of protein acetylation sites using a logarithmic conversion with a base of 2 (log2). Next, we selected proteins with a standard deviation > 0.3 to ensure substantial variation. After this screening process, we identified 432 modified proteins for further analysis. We utilized the Mfuzzy method to cluster the expression patterns of modified proteins. The clustering parameters included six clusters (k) and a fuzzy degree (m) of 2. Notably, Cluster1, Cluster2, and Cluster3 exhibited distinctive patterns that reflected the impact of caffeine treatment, indicating its potential to improve acetylation modification of lysine in the white matter of HI neonatal rats.

We performed GO function, KEGG pathway, and protein domain enrichment analyses for the proteins in each cluster (Figure 3D). The results revealed that Cluster1 was predominantly enriched in the mitochondrial matrix and secondary endosomes, with protein domains including isopropyl citrate/isopropyl malate dehydrogenase and hexokinase. Its function was closely related to nicotinamide adenine dinucleotide (NAD) binding, coenzyme binding, cytokine production, and positive regulation of cytokine secretion. Many functions and pathways were linked to propionate metabolism and lysine degradation, which are crucial for mitochondrial activity. Cluster2 was mainly localized in cell junctions and intercellular connections, with SH2 and SH3 protein domains. Its functions and pathways were related to gap linking, cell entry, white cell migration, axon guidance, insulin receptor binding, transferase activity, and glycosyl transfer. Cluster3 played a role in the nuclear and inner mitochondrial membrane protein complexes, with specialist repeats and Ca^2+^-sensitive EF hand protein domains. Its functions and pathways were related to thermogenic effects, oxidative phosphorylation, isomerase activity, and silk protein-based movement. These pathways were consistent with the KEGG pathway and IPA analyses of differentially expressed proteins, suggesting that caffeine improves mitochondrial dysfunction through protein acetylation, a concept which requires further exploration.

Further examination of acetylation's role post-caffeine treatment in neonatal rats with HI-induced brain damage involved mapping the protein interactions within three clusters. In this comprehensive network, we included 159 acetylation sites and 133 proteins, assessing the interactions with a stringent threshold. The resulting network visualization displayed the regulatory patterns of these proteins in response to treatment (Figure 3E).

To characterize the complexes between acetylated proteins, 133 proteins from Cluster1, Cluster2, and Cluster3 were used as protein interaction networks. The acetylated modified proteins related to the citric acid cycle, tricarboxylic acid cycle, mitochondrial phagocytosis, oxidative phosphorylation, inner mitochondrial membrane proteins, mitochondrial organelles, and other keywords were classified by clustering.

Upon additional analysis, we found that compared to the HI group, Mapt was downregulated in both the Sham group and the Caffeine group. This gene is significantly differentially expressed and is closely related to mitochondrial function. Further verification of this finding is warranted in future investigations.

### 3.4 Caffeine improved mitochondrial function in neonatal rats after HI-induced WMD

**Figure 4:**
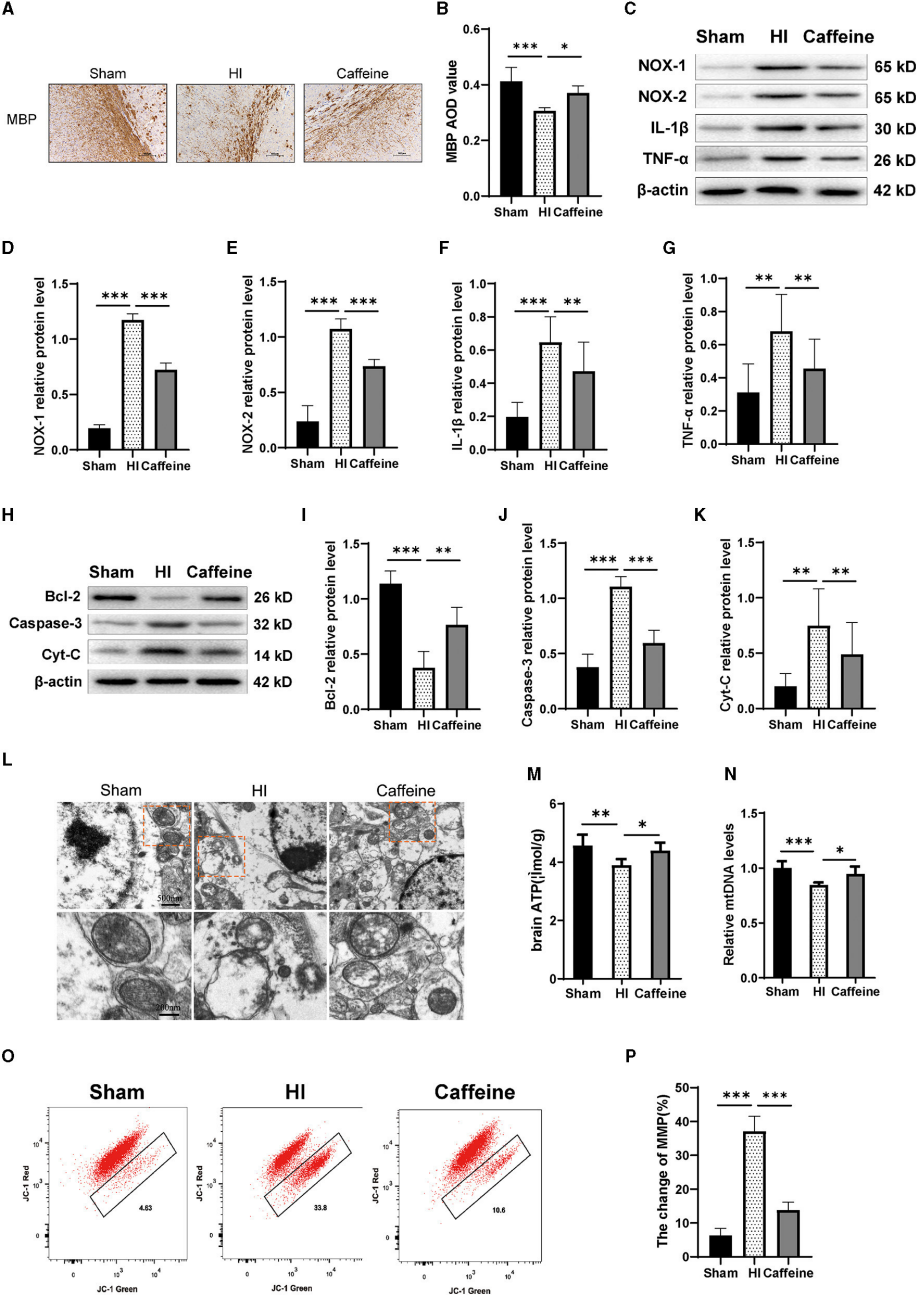
Caffeine improves mitochondrial function after hypoxia-ischemia (HI)-induced white matter damage in neonatal rats. **(A)** Immunohistochemical MBP staining in the corpus callosum (CC) on the ligated side of the cerebral hemisphere at 14 days after hypoxic-ischemia (MBP scale bar = 100 μm). **(B)** Average optical density (AOD) values of MBP staining. **(C)** Western blot detection of NOX-1, NOX-2, IL-1β, and TNF-α. Analyses of relative **(D)** NOX-1, **(E)** NOX-2, **(F)** IL-1β, and **(G)** TNF-α levels, with β-actin used for normalization. **(H)** Western blot detection of Bcl-2, Caspase-3 and cytochrome C (Cyt-C). **(I)** Analyses of relative Bcl-2, **(J)** Caspase-3, and **(K)** Cyt-C levels, with β-actin used for normalization. **(L)** Electron microscopy was performed to evaluate mitochondrial structure and function. Scale bar = 500 nm. Enlarged scale bar = 200 nm. **(M)** ELISA was used to determine ATP levels. **(N)** Polymerase chain reaction (PCR) analysis was employed to determine mitochondrial DNA (mtDNA) levels. Levels were normalized against those of glyceraldehyde 3-phosphate dehydrogenase (Gapdh) and expressed as the fold change. **(O)** Mitochondrial Membrane Potential (MMP) assay. **(P)** Change rate of MMP. Data represent the mean ± standard error of the mean. Statistical analyses involve one-way ANOVA. *p < 0.05, **p < 0.01, ***p < 0.001. Sham group (*n* = 6); HI group (*n* = 6); Caffeine group (*n* = 6).

In previous research, we noted alterations in histone acetylation following caffeine treatment in neonatal rats with HI-induced damage. We then extended our study to assess caffeine's impact on oxidative stress and inflammation, as well as its effect on mitochondria in this context. Myelination status was gauged using MBP staining, which revealed disarray in nerve fiber organization and reduced myelination in the HI group (*P* < 0.001) (Figure 4A), with some recuperation observed in the Caffeine group (*P* < 0.05) (Figure 4B).

Western blotting analysis were conducted to measure the expression levels of NOX-1, NOX-2, IL-1β, and TNF-α proteins. Our results showed that compared to the Sham group, the levels of NOX-1, NOX-2, IL-1β, and TNF-α proteins were increased in the HI group. However, administration of caffeine reduced the levels of these proteins following HI-induced WMD (Figures 4C–G, *p* < 0.01 or *p* < 0.001).

Next, we detected the levels of apoptotic proteins Caspase-3 and Bcl-2. The results showed that the HI group exhibited a significant increase in Caspase-3 protein levels compared to the Sham group. However, caffeine reduced Caspase-3 protein levels after WMD. On the contrary, compared with the Sham group, the HI group had a decrease in Bcl-2 protein levels, while the Caffeine group had an improvement in Bcl-2 protein levels (Figures 4H–J, *p* < 0.01 and *p* < 0.001, respectively). Then we examined the structure and function of the mitochondria to evaluate the role of caffeine in rescuing damaged mitochondria. Cytochrome C (Cyt-C) levels, used as an indicator of mitochondrial damage, were analyzed in tissues, and the results showed that the HI group exhibited a significant increase in Cyt-C levels compared to the Sham group. However, caffeine reduced Cyt-C levels after HI-induced WMD (Figures 4H, K, *p* < 0.01). The mitochondria were observed by transmission electron microscopy. In the Sham group, the mitochondria had a distinct cristae appearance. Following HI, the mitochondria showed abnormalities including swelling, ridge collapse, and membrane rupture in the HI group. Importantly, caffeine treatment increased the mitochondrial tubular network and maintained normal mitochondrial morphology (Figure 4L). Similar results were observed in terms of improving mitochondrial dysfunction, including ATP and mitochondrial DNA (mtDNA) levels. The results showed that the HI group reduced in ATP and mDNA levels compared to the Sham group. However, caffeine increased ATP and mDNA levels (Figures 4M, N, *p* < 0.05, *p* < 0.01, and *p* < 0.001, respectively). The decrease in cell membrane potential can be easily detected through the transition of JC-1 from red fluorescence to green fluorescence. In normal cells, this dye accumulates and aggregates in mitochondria, forming bright red fluorescent clusters. When the membrane potential of the cell mitochondria decreases, the red color changes to green. Therefore, we observed a decrease in membrane potential in the HI group and an improvement in membrane potential in the Caffeine group compared to the Sham group (Figures 4O, P, *p* < 0.001).

### 3.5 Caffeine promoted the effects of deacetylation after HI-induced WMD in neonatal rats

**Figure 5:**
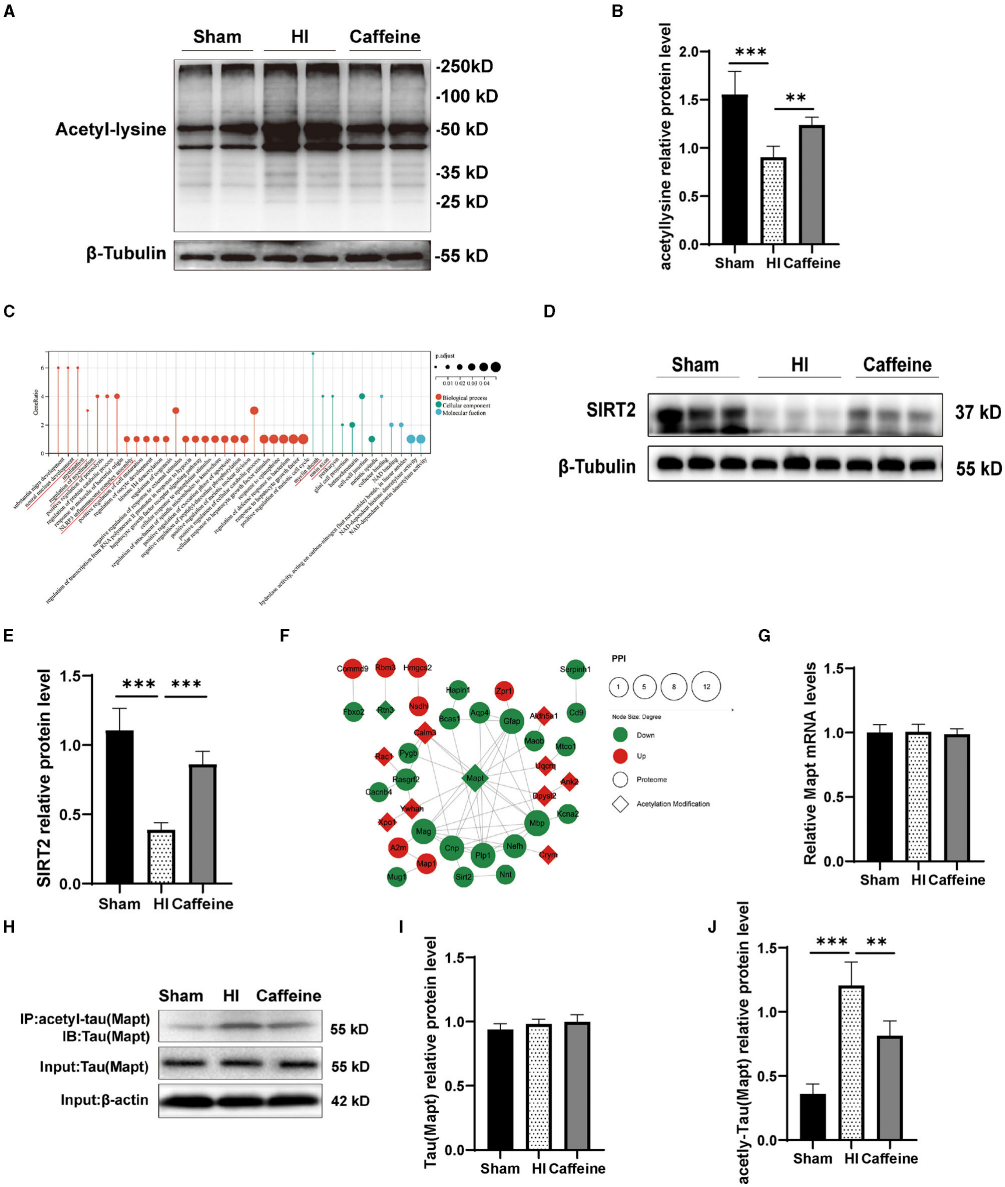
Caffeine promoted the effects of deacetylation after hypoxia-ischemia-induced WMD in neonatal rats. **(A)** Western blots of proteins in the white matter of brains of neonatal rats on day 14 using an anti-acetylated-lysine antibody. **(B)** Analyses of relative acetylated-lysine levels, with β-tubulin used for normalization. **(C)** Gene Ontology (GO) of SIRT2. **(D)** Western blot detection of SIRT2. **(E)** Relative protein level of SIRT2. **(F)** Analysis of the protein-protein interaction (PPI) networks of differential acetylation modification and proteome protein. **(G)** Polymerase chain reaction (PCR) analysis to determine mRNA levels of Mapt. Mapt levels are normalized against those of glyceraldehyde 3-phosphate dehydrogenase (GADPH) and expressed as the fold change. **(H)** Mapt immunoprecipitation with anti-acetyl-lysine antibody and analysis with anti-tau. **(I)** Analyses of relative total Mapt levels, with β-actin used for normalization. **(J)** Analyses of relative acetyl-Mapt levels, with Mapt used for normalization. Data is presented as the mean ± standard error of the mean. Statistical analyses involved one-way ANOVA. The artwork with “^*^” indicate statistically significant differences between groups. ^*^p < 0.05, ^**^p < 0.01, ^***^p < 0.001. Sham group (*n* = 6); HI group (*n* = 6); Caffeine group (*n* = 6); Caffeine+AK-7 group (*n* = 6).

To ensure the accuracy of our proteomic analysis of lysine acetylation, we conducted a Western blot analysis to confirm the overall acetylation modifications of the three groups. The results showed differences in acetylated proteins among the Sham, HI, and Caffeine groups. Specifically, the HI group had an increased rate of lysine acetylation near a molecular weight of 50 KD compared to the Sham and Caffeine groups (Figures 5A, B, *p* < 0.01 or *p* < 0.001). This suggests that caffeine may have a positive effect on protein deacetylation during HI-induced WMD.

According to our previous research, proteomic analysis identified 47 proteins that were differentially expressed among the Sham, HI, and Caffeine groups. These proteins are potentially linked to SIRT2-mediated myelin development and synaptic formation (Yang et al., 2022a) (Figure 5C). SIRT2 is an enzyme known as a nicotinamide adenine dinucleotide-dependent deacetylase. SIRT2, a key player in the myelination process and implicated in various neurological conditions, was scrutinized (Fourcade et al., 2017; Chen et al., 2021). Western blot analysis confirmed a decrease in SIRT2 expression in the HI group (*p* < 0.001), with a marked increase observed in the Caffeine group, suggesting a potential protective or restorative effect of caffeine (*p* < 0.001) (Figures 5D, E).

Cluster analysis revealed that the acetylated modified proteins in Cluster3 primarily participate in the protein complex of the inner mitochondrial membrane. Within Cluster3, Mapt exhibited significant positive changes in deacetylation. Through the PPI analysis of acetylated and proteomic proteins (Yang et al., 2022a), we found that there is a protein interaction between SIRT2 and Mapt (Figure 5F). Therefore, we applied WB and PCR to validate the levels of full and acetylated Mapt proteins. The results showed that Mapt mRNA levels did not differ among the three groups (Figure 5G). The acetylation level of Mapt protein in the HI group was significantly upregulated compared to that in the Sham group, and this increase was observed to be inhibited in the Caffeine group (Figures 5H, J, *p* < 0.01 or *p* < 0.001). However, there was no difference in total Mapt protein levels among the three groups (Figures 5H, I). This result is consistent with findings from studies analyzing acetylated proteins using multiphoton spectroscopy, which further verifies the accuracy and feasibility of omics research results.

### 3.6 Caffeine deacetylated Mapt through SIRT2 after HI-induced WMD in neonatal rats

To further investigate the relationship between Mapt deacetylation, SIRT2, and the prevention and treatment of caffeine in WMD, we conducted experiments on neonatal rats. The rats were treated with caffeine and the SIRT2 inhibitor AK-7 to evaluate changes in Mapt acetylation in WMD, as well as their impact on mitochondrial function. Western blot analysis was performed to measure the levels of total Mapt, acetylated Mapt, Cyt-C, mitochondrial transcription factor A (TFAM), Caspase-3 and Bcl-2 proteins.

**Figure 6:**
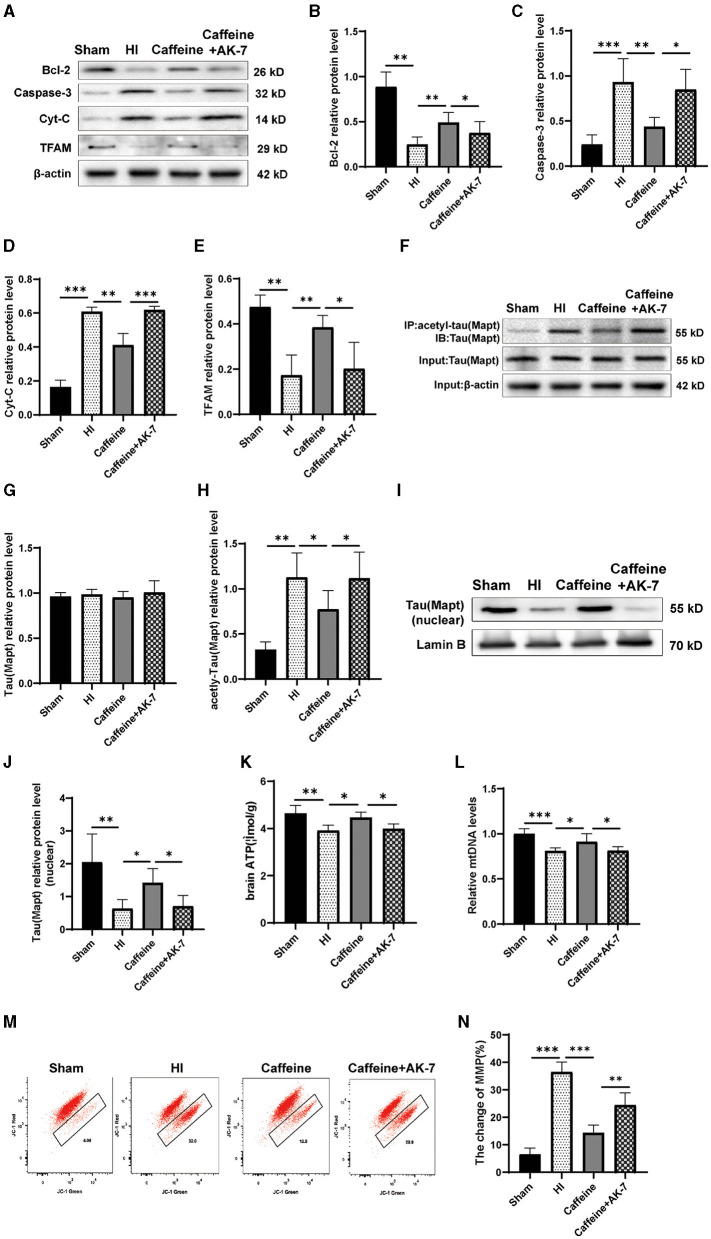
Caffeine deacetylated Mapt through SIRT2 after hypoxia-ischemia-induced WMD in neonatal rats. **(A)** Western blot detection of Bcl-2, Caspase-3, cytochrome C (Cyt-C) and mitochondrial transcription factor A (TFAM). **(B)** Analyses of relative Bcl-2, **(C)** Caspase-3, **(D)** Cyt-C, and **(E)** TFAM levels, with β-actin used for normalization. **(F)** Mapt was immunoprecipitated with anti-acetyl-lysine antibody and analyzed with anti-tau. **(G)** Analyses of relative total Mapt levels, with β-actin used for normalization. **(H)** Analyses of relative acetyl-Mapt levels, with Mapt used for normalization. **(I)** Western blot detection of nuclear Mapt. **(J)** Analyses of relative nuclear Mapt protein levels, with Lamin B used for normalization. **(K)** ELISA to determine ATP levels. **(L)** Polymerase chain reaction (PCR) analysis to determine mitochondrial DNA (mtDNA) levels. Levels are normalized against those of glyceraldehyde 3-phosphate dehydrogenase (GADPH) and are expressed as the fold change. **(M)** Mitochondrial Membrane Potential (MMP) assay. **(N)** Change rate of MMP. Data is presented as the mean ± standard error of the mean. Statistical analyses involve one-way ANOVA. The artwork with “*” indicate statistically significant differences between groups. *p < 0.05, **p < 0.01, ***p < 0.001. Sham group (*n* = 6); HI group (*n* = 6); Caffeine group (*n* = 6); Caffeine+AK-7 group (*n* = 6).

The results revealed that when AK-7 was applied, the levels of Cyt-C and Caspase-3 in the caffeine+AK-7 group were significantly increased compared to those in the caffeine-only group (Figures 6A, C, D, *p* < 0.05 or *p* < 0.001). Compared with the Sham group, TFAM and Bcl-2 protein levels were downregulated after HI and upregulated after caffeine treatment, but this improvement was inhibited after the application of AK-7 (Figures 6A, B, E, *p* < 0.01 or *p* < 0.05). Moreover, the degree of acetylation of the Mapt protein was also upregulated in the caffeine+AK-7 group (Figures 6F–H, *p* < 0.05). Meanwhile, compared with the Sham group, Mapt nuclear translocation was reduced after HI. Caffeine treatment can promote nuclear translocation, but this effect is inhibited after the application of AK-7 (Figures 6I, J, *p* < 0.05 or *p* < 0.01). Similar findings were observed in terms of rescuing mitochondrial dysfunction. When AK-7 was applied, the caffeine+AK-7 group exhibited a decrease in ATP and mtDNA levels compared to the Caffeine group (Figures 6K, L, *p* < 0.05). MMP detection revealed a decrease in membrane potential in the Caffeine+AK-7 group compared to the Caffeine group (Figures 6M, N, *p* < 0.01). The effect of caffeine on improving membrane potential is inhibited by AK-7.

## 4 Discussion

Lysine acetylation, a widespread and evolutionary conserved modification that takes place after protein synthesis, is involved in numerous cellular roles (Choudhary et al., 2009). In this study, we employed affinity enrichment and HPLC-MS/MS analyses to investigate the overall changes in acetylation in the white matter of rats in Sham surgery, brain injury, and caffeine treatment groups. A total of 1999 lysine acetylation sites were identified among 1,123 proteins. To gain insights into the functions of these acetylated proteins, a thorough bioinformatics analysis was conducted.

The distribution pattern of lysine acetylation sites observed in our study within proteins is mainly coil configurations, followed by α-helix and β-strand regions, might reflect a functional aspect of protein structure relevant to post-translational modifications. Protein regions that are in coil configurations tend to be more accessible and flexible, making them prime candidates for modifications such as acetylation. Acetylation at sites within helical regions of proteins may precipitate alterations in their structural integrity and functional capacity. This post-translational modification typically attenuates the positive charge on proteins, potentially diminishing their electrostatic interactions with a spectrum of biomolecules. Such a reduction in electrostatic affinity may consequently impair the protein's capacity for binding to other proteinaceous entities or nucleic acid structures (Castro et al., 2023). In the context of WMD, this structural change may affect the signal transduction and metabolic pathways of nerve cells, leading to neurological deficits and further development of nerve damage.

In neonatal rats afflicted with WMD that have been treated with caffeine, we found that acetylation patterns in proteins are linked to a variety of cellular functions, with a notable emphasis on mitochondrial activities. Mitochondria are versatile and critical for energy generation, calcium regulation, signal transduction, and cell survival, particularly during injury and pathological conditions, constantly undergoing changes to fulfill their crucial function. Recent studies have shed light on the diverse functions of mitochondria in neuropathy, neurodegeneration, and immune activation, both in normal and abnormal settings. In the early stages of brain injury, mitochondrial dysfunction manifests as oxidative stress, inflammation, bioenergy deficiency, impaired biogenesis and transport, and disrupted autophagy processes (Soustiel and Larisch, 2010; Vongsfak et al., 2021).

Our investigation into the functional landscape altered by acetylation modifications has revealed a diverse array of biological processes that are influenced by these changes. Among them, the regulation of cell size and the intricate development of the central nervous system stand out as particularly crucial. Noteworthy is the fact that these processes are vital for the generation of action potentials, suggesting a profound impact of acetylation on neuronal signaling and communication (Jacob et al., 2011; Marchi et al., 2019). The analysis further identified a notable enrichment of acetylated proteins within key cellular components such as the primary endosome membrane, neuronal axons, intervertebral discs, and cell junction regions, highlighting the widespread influence of acetylation on cellular architecture and integrity (Eshun-Wilson et al., 2019). This spatial enrichment suggests that acetylation may play a localized regulatory role, potentially affecting cellular trafficking, signal transduction, and structural stability (Jeong and Cho, 2017). In terms of molecular functionality, our results underscored a significant enrichment in molecular functions essential for cellular homeostasis and communication, including GTP-dependent protein binding, ATPase activity, and generic protein interactions. These findings indicate that acetylation may regulate critical aspects of molecular dynamics and energy utilization within the cell, which could have far-reaching implications for cellular metabolism and signaling pathways by mitochondria. Our foray into the KEGG pathways via functional enrichment analysis illuminated the substantial involvement of specific pathways, particularly those related to neurotrophin and PI3K-Akt signaling. The congruence of these pathways with the acetylation patterns we initially uncovered not only validates our proteomic results but also suggests a possible regulatory mechanism by which acetylation modulates signal transduction pathways critical for neuronal survival, plasticity, and growth (Liu et al., 2018; Zhu et al., 2018).

When serious diseases occur, the association between changes in acetylation sites and the disease may be more complex. On the one hand, acetylation can regulate different signaling pathways and protein complexes, and incorrect acetylation levels may lead to the development of diseases such as tumors and neurodegenerative diseases. Based on this, changes in the number of bits may be involved in the pathological process of the disease. However, the number of acetylation modifications does not directly correlate with protein abundance, making it difficult to make quantitative predictions about changes in the number of acetylation sites (Yang W. et al., 2022). On the contrary, the specificity of acetylation sites and their environment in disease-related proteins may be more critical factors determining their impact on disease severity. So, our research indicates that the number of acetylation sites in each protein is mostly 2–3. Considering the changes in the number of acetylation sites in each protein, there may be a certain correlation between the specific protein's function and the severity of WMD. However, this correlation is not a simple proportional relationship, but is determined by the protein's function, its position in the cell, and its biological background. To gain a deeper understanding of this association, more detailed functional studies are needed at the protein level and the neuroprotective effects of caffeine

Caffeine, which counteracts adenosine receptors, is known for its protective attributes, including antioxidative, anti-inflammatory, and cell death prevention capabilities. Additionally, caffeine exhibits free radical scavenging capabilities and is a multifunctional neuroprotective drug in the developing brain. Studies have shown that it not only has a protective effect on hypoxic-ischemic brain injury in premature infants, but also has a protective effect on other forms of brain injury in newborns, such as hyperoxia induced brain injury and traumatic brain injury (Endesfelder et al., 2017; Lusardi et al., 2020; Heise et al., 2023). This potential positions caffeine as an intriguing candidate for therapeutic strategies designed to ameliorate the adverse consequences of mitochondrial dysfunction in WMD (Lusardi et al., 2020; Heise et al., 2023) and to mitigate oxidative damage to mitochondria (Kamat and Devasagayam, 2000). Recent studies have demonstrated that caffeine can induce mitochondrial fission by activating the cAMP/PKA/AMPK signaling pathway. This pathway plays a role in regulating mitochondrial dynamics in endothelial cells. Additionally, caffeine has been found to influence endothelial mitochondrial dynamics and promote angiogenesis, the formation of new blood vessels. These findings suggest that caffeine may have potential therapeutic implications for conditions involving impaired mitochondrial function and disrupted endothelial dynamics (Wang et al., 2021). Caffeine has been shown to potentially reduce oxidative damage triggered by brain mitochondria and lower the seizure threshold in conditions associated with tricuspid valve regurgitation-induced seizures. This suggests that caffeine may exert a protective effect on brain mitochondria, potentially minimizing the harmful effects of oxidative stress and thereby reducing the likelihood of seizures occurring (Samadi et al., 2021).

Our investigation presents compelling evidence for the involvement of site-specific lysine acetylation modifications in the physiological and pathological processes of diverse diseases. Notably, we identified alterations in protein acetyl groups within mitochondria, underscoring the pivotal role of lysine acetylation in modulating mitochondrial functionality. Emerging research suggests the neuroprotective action of caffeine may stem from its capacity to attenuate oxidative stress and neuroinflammation (Ösz et al., 2022; Tiwari et al., 2023), likely through the regulation of acetylation modifications. This, in turn, enhances mitochondrial performance, thus mitigating oxidative and inflammatory damage. Our preceding studies have further verified caffeine's ability to modulate microglial phenotype polarization, thereby exerting an anti-neuroinflammatory effect (Yang et al., 2022b). In the current study, we observed a reduction in oxidative stress markers, such as NOX-1 and NOX-2, in the caffeine-treated cohort, alongside a decrease in inflammatory cytokines including IL-1β and TNF-α. These findings indicate caffeine's potential to diminish brain oxidative stress and inflammation. Moreover, transmission electron microscopy revealed that caffeine treatment significantly mitigated mitochondrial damage compared to the HI group, evidenced by reduced Cyt-C levels and increased ATP and mDNA concentrations. Collectively, these results demonstrate caffeine's efficacy in enhancing mitochondrial function in neonatal rats with WMD, providing a promising therapeutic avenue for addressing mitochondrial dysfunctions.

The data we collected implies that caffeine may contribute to a decrease in oxidative stress and inflammation in the brain, while bolstering mitochondrial efficiency in neonatal rats experiencing HI WMD. Additional investigations have identified variances in the acetylation of the Mapt protein among the groups studied, which plays a part in the protein assembly inside the mitochondrial inner membrane.

Mapt is a protein primarily expressed in neurons of the central nervous system and is involved in various microtubule-related functions, serving as a vital component of the neuronal cytoskeleton (Avila et al., 2004). In neurons, Mapt, after post-translational modification, not only regulates the stable transport and distribution of mitochondria (Korn et al., 2023), but may also affect the energy production and release of mitochondria through microtubule networks, as well as the apoptosis and survival processes of neurons (Caceres and Kosik, 1990; Hartmann et al., 2024). Studies have indicated that excessive acetylation of Mapt can impede its depolymerisation process. This impairment leads to the accumulation of abnormal Mapt in cells, which can have cytotoxic effects on neuronal function and viability (Cohen et al., 2011; Trzeciakiewicz et al., 2017; Ferreon et al., 2018). Recent studies have demonstrated that elevated levels of Mapt acetylation can impact other post-translational modifications. One notable effect is the inhibition of phosphorylated Mapt degradation, resulting in its accumulation within cells. This aberrant accumulation of phosphorylated Mapt can have significant implications for cellular function and contribute to pathological processes (Min et al., 2018; Shi et al., 2021). Excessive phosphorylation or acetylation of Mapt has been associated with neurotoxicity and is believed to potentially contribute to neurodevelopmental disorders in infants (Sarnat and Flores-Sarnat, 2015), and has been strongly linked to neurodegenerative disorders (Zhang et al., 2016; Toker et al., 2021; Ruiz-Gabarre et al., 2022). The role of Mapt in ischemic brain injury has garnered considerable interest, primarily due to observations of abnormally high acetylation levels of Mapt during ischemic brain injury which is associated with neurological impairment and cerebral infarction (Shi et al., 2021). Our investigation delineated and validated alterations in Mapt protein acetylation across three distinct study cohorts, while total protein levels remained unchanged. Intriguingly, we observed an increase in acetylated Mapt protein in the HI group, concomitant with a reduction in its nuclear presence. This suggests that post-injury, the excessive acetylation of intracellular Mapt may impede its nuclear translocation, thereby obstructing its mitochondrial functionality.

Mitochondrial proteins undergo extensive acetylation, especially in energy-producing mitochondria, where the average number of acetylation sites is relatively high. The main regulatory mechanism for mitochondrial deacetylation involves NAD-dependent deacetylases known as sirtuins (He et al., 2012; Hebert et al., 2013; Baeza and Smallegan, 2016). Research has revealed that SIRT1 plays a specific role in deacetylating Mapt at specific lysine residues. These residues are likely located within the regions spanning positions 160–182 and 264–287 of the Mapt protein (Min et al., 2010, 2018; Shi et al., 2021). Modulating the SIRT1/Mapt pathway could potentially have a significant impact on stroke recovery and cerebral ischemia/reperfusion function. Another member of the sirtuin family, SIRT2, is closely associated with Mapt in the context of HIV-related neurological impairment (Duran-Castells et al., 2023). Studies have suggested a link between SIRT2 and Mapt, a key protein associated with neurodegeneration, during whole-brain radiotherapy (Shukla et al., 2015). Our acetylomics IPA analysis has revealed that acetylation is associated with neuroinflammatory signaling pathways. Additionally, SIRT2 has been found to be closely associated with pathological processes including neuroinflammation, axonal synaptic dysfunction, metabolic abnormalities, and oxidative stress (Wang et al., 2016; Shu et al., 2019).

**Figure 7:**
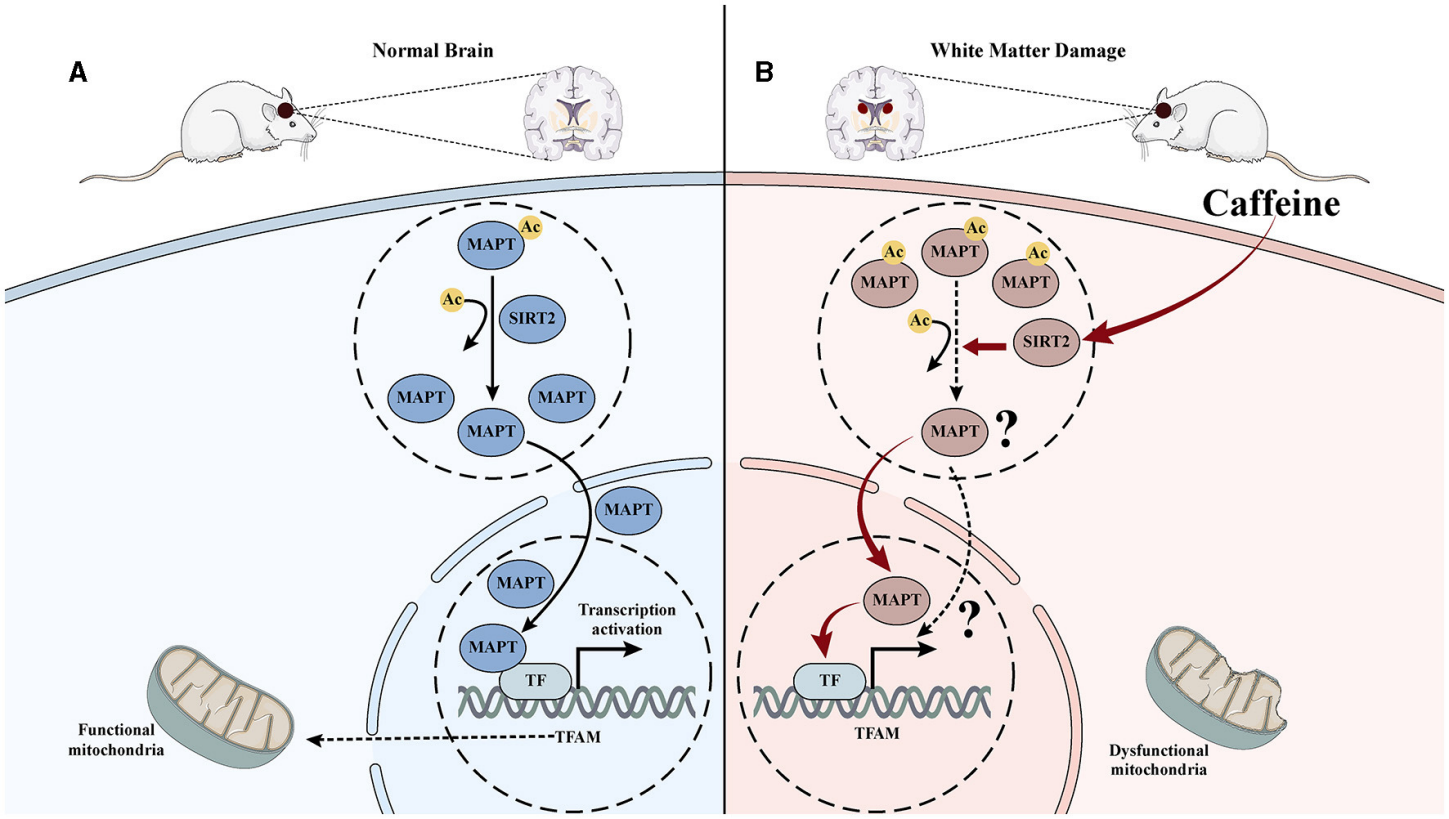
Mechanism diagram of caffeine inhibiting Mapt acetylation by upregulating SITR2, promoting its nuclear translocation, and improving mitochondrial damage. **(A)** In normal brain tissue, Mapt is deacetylated and translocated into the nucleus, thereby promoting mitochondrial transcription in the nucleus and maintaining mitochondrial function. **(B)** After hypoxia-ischemia-induced WMD, the acetylation level of Mapt increased, nuclear translocation decreased, mitochondrial transcription decreased, and damage increased. Induced to caffeine treatment, SIRT2 was activated, Mapt deacetylation increased, nuclear translocation increased, mitochondrial transcription increased, and damage improved.

Our previous proteomics research has successfully identified and validated SIRT2 as a crucial target for caffeine in exerting brain-protective effects in HI WMD (Yang et al., 2022a). In the current study, we discovered a protein interaction between SIRT2 and Mapt using PPI analysis. We also observed that inhibiting SIRT2 led to reduced deacetylation of Mapt, reduced nuclear translocation and diminished effectiveness of caffeine in improving mitochondrial damage. Overall, these findings suggest that the mechanism behind caffeine mediated SIRT2 deacetylation modification of Mapt may be associated with mitochondrial function (Figures 7A, B).

This study faced certain limitations. Firstly, due to the absence of commercially available antibodies targeting specific acetylation sites, we were only able to validate the results using pan acetylation antibodies without identifying the precise sites. There was no further exploration of the effect of caffeine on mitochondrial protein acetylation, and there was a lack of specific pathways. In the future, continuous elucidation of specific proteins and precise lysine residues acetylated within mitochondria will deepen our understanding of how acetylation affects neuronal mitochondrial function. Secondly, previous studies have suggested a link between Mapt acetylation and phosphorylation, indicating a potential dynamic balance in post-translational modifications (Cohen et al., 2011; Trzeciakiewicz et al., 2017; Ferreon et al., 2018). In our study, we primarily focused on examining the association between caffeine, SIRT2, and the acetylation status of Mapt. We did not investigate the phosphorylation changes of Mapt or explore the interaction mechanisms between different post-translational modifications. These aspects will be addressed in our future research. Moreover, while we observed that SIRT2 deacetylation of Mapt might be linked to mitochondrial function, the specific molecular mechanism remains unclear and requires further investigation.

## 5 Conclusions

Through affinity enrichment and HPLC-MS/MS, our work uncovered novel insights into acetylated proteins in the white matter region of neonatal rats and the impact of caffeine treatment on rats with WMD. The results suggested that caffeine may inhibit acetylated Mapt via SIRT2, leading to an improvement in mitochondrial dysfunction. These findings hold significant implications for future research and propose a promising treatment approach for WMD in newborns.

## Data availability statement

The data that support the findings of this study are available from the corresponding author upon reasonable request.

## Ethics statement

All animal experiments were approved by the Animal Ethics Committee of China Medical University, Shenyang, China (approval no. 2021PS839K) and we were in accordance with the 1975 Helsinki declaration and its later amendments. The study was conducted in accordance with the local legislation and institutional requirements.

## Author contributions

YZ: Formal analysis, Methodology, Writing—original draft. YW: Conceptualization, Investigation, Software, Writing—original draft. HD: Conceptualization, Data curation, Writing—original draft. SW: Data curation, Methodology, Writing—review & editing. DQ: Data curation, Methodology, Writing—review & editing. XP: Data curation, Methodology, Writing—review & editing. NZ: Methodology, Writing—review & editing. LY: Conceptualization, Funding acquisition, Writing—review & editing.

## References

[B1] Arduíno D. M. Esteves A. R. Oliveira C. R. Cardoso S. M. (2010). Mitochondrial metabolism modulation: a new therapeutic approach for Parkinson's disease. CNS Neurol. Disord Drug Targets 9, 105–119. 10.2174/187152710790966687 20201821

[B2] Avila J. Lucas J. J. Perez M. Hernandez F. (2004). Role of tau protein in both physiological and pathological conditions. Physiol. Rev. 84, 361–384. 10.1152/physrev.00024.2003 15044677

[B3] Baeza J. Smallegan M. J. (2016). Denu JMechanisms M, and dynamics of protein acetylation in mitochondria. Trends Biochem. Sci. 41, 231–244. 10.1016/j.tibs.2015.12.006 26822488 PMC4783225

[B4] Bano S. Chaudhary V. Garga U. C. (2017). Neonatal hypoxic-ischemic encephalopathy: a radiological review. J. Pediatr. Neurosci. 12, 1–6. 10.4103/1817-1745.205646 28553370 PMC5437770

[B5] Caceres A. Kosik K. S. (1990). Inhibition of neurite polarity by tau antisense oligonucleotides in primary cerebellar neurons. Nature 343, 461–463. 10.1038/343461a0 2105469

[B6] Castro T. G. Ferreira T. Matam,á T. Munteanu F. D. Cavaco-Paulo A. (2023). Acetylation and phosphorylation processes modulate Tau's binding to microtubules: a molecular dynamics study. Biochim. Biophys. Acta Gen. Subj. 1867:130276. 10.1016/j.bbagen.2022.130276 36372288

[B7] Chamberlain K. A. Huang N. Xie Y. LiCausi F. Li S. Li Y.. (2021). Oligodendrocytes enhance axonal energy metabolism by deacetylation of mitochondrial proteins through transcellular delivery of SIRT2. Neuron 109, 3456–3472.e8. 10.1016/j.neuron.2021.08.011 34506725 PMC8571020

[B8] Chen X. Lu W. Wu D. (2021). Sirtuin 2 (SIRT2): confusing roles in the pathophysiology of neurological disorders. Front. Neurosci. 15:614107. 10.3389/fnins.2021.614107 34108853 PMC8180884

[B9] Cheng T. Xue X. Fu J. (2015). Effect of OLIG1 on the development of oligodendrocytes and myelination in a neonatal rat PVL model induced by hypoxia-ischemia. Mol. Med. Rep. 11, 2379–2386. 10.3892/mmr.2014.3028 25435330 PMC4337744

[B10] Chou M. F. Schwartz D. (2011). Biological sequence motif discovery using motif-x. Curr Protoc Bioinformatics. Chapter 13, 13.15.1–13.15.24. 10.1002/0471250953.bi1315s35 21901740

[B11] Choudhary C. Kumar C. Gnad F. Nielsen M. L. Rehman M. Walther T. C.. (2009). Lysine acetylation targets protein complexes and co-regulates major cellular functions. Science 325, 834–840. 10.1126/science.1175371 19608861

[B12] Cohen T. J. Guo J. L. Hurtado D. E.. (2011). The acetylation of tau inhibits its function and promotes pathological tau aggregation. Nat. Commun. 2, 252. 10.1038/ncomms1255 21427723 PMC3120096

[B13] Cooper M. S. Mackay M. T. Fahey M. Reddihough D. Reid S. M. Williams K.. (2017). Seizures in children with cerebral palsy and white matter injury. Pediatrics. 139:e20162975. 10.1542/peds.2016-2975 28209769

[B14] Ding Q. Xu Y. M. Lau A. (2023). The epigenetic effects of coffee. Molecules. 28:1770. 10.3390/molecules28041770 36838754 PMC9958838

[B15] Duran-Castells C. Llano A. Kawana-Tachikawa A. Prats A. Martinez-Zalacain I. Kobayashi-Ishihara M.. (2023). Sirtuin-2, NAD-dependent deacetylase, is a new potential therapeutic target for HIV-1 infection and HIV-related neurological dysfunction. J. Virol. 97:e0165522. 10.1128/jvi.01655-22 36719240 PMC9972991

[B16] Endesfelder S. Weichelt U. Strau,ß E. Schlör A. Sifringer M. Scheuer T.. (2017). Neuroprotection by caffeine in hyperoxia-induced neonatal brain injury. Int. J. Mol. Sci. 18:187. 10.3390/ijms18010187 28106777 PMC5297819

[B17] Eshun-Wilson L. Zhang R. Portran D. Nachury M. V. Toso D. B. Löhr T.. (2019). Effects of α-tubulin acetylation on microtubule structure and stability. Proc. Natl. Acad. Sci. U.S.A. 116, 10366–10371. 10.1073/pnas.1900441116 31072936 PMC6535015

[B18] Ferreon J. C. Jain A. Choi K.-J. Tsoi P. S. MacKenzie K. R. Jung S. Y.. (2018). Acetylation disfavors tau phase separation. Int. J. Mol. Sci. 19. 10.3390/ijms19051360 29734651 PMC5983838

[B19] Fleiss B. Van Steenwinckel J. Bokobza C. Shearer K. Ross-Munro E. Gressens P. (2021). Microglia-mediated neurodegeneration in perinatal brain injuries. Biomolecules. 11. 10.3390/biom11010099 33451166 PMC7828679

[B20] Fourcade S. Morat,ó L. Parameswaran J. Ruiz M. Ruiz-Cortés T. Jov,é M.. (2017). Loss of SIRT2 leads to axonal degeneration and locomotor disability associated with redox and energy imbalance. Aging Cell. 16, 1404–1413. 10.1111/acel.12682 28984064 PMC5676070

[B21] Hartmann C. Anskat M. Ehrlich M. Sterneckert J. Pal A. Hermann A.. (2024). Mapt mutations V337M and N297K alter organelle trafficking in frontotemporal dementia patient-specific motor neurons. Biomedicines 12:641. 10.3390/biomedicines12030641 38540253 PMC10968393

[B22] He W. Newman J. C. Wang M. Z. Ho L. Verdin E. (2012). Mitochondrial sirtuins: regulators of protein acylation and metabolism. Trends Endocrinol. Metab. 23, 467–476. 10.1016/j.tem.2012.07.004 22902903

[B23] Hebert A. S. Dittenhafer-Reed K. E. Yu W. Bailey D. J. Selen E. S. Boersma M. D.. (2013). Calorie restriction and SIRT3 trigger global reprogramming of the mitochondrial protein acetylome. Mol. Cell. 49, 186–199. 10.1016/j.molcel.2012.10.024 23201123 PMC3704155

[B24] Heise J. Schmitz T. Bührer C. Endesfelder S. (2023). Protective effects of early caffeine administration in hyperoxia-induced neurotoxicity in the juvenile rat. Antioxidants (Basel) 12:295. 10.3390/antiox12020295 36829854 PMC9952771

[B25] Horton P. Park K. J. Obayashi T. Fujita N. Harada H. Adams-Collier C. J.. (2007). WoLF PSORT: protein localization predictor. Nucleic Acids Res. 35, W585–W587. 10.1093/nar/gkm259 17517783 PMC1933216

[B26] Huntley M. A. Golding G. B. (2002). Simple sequences are rare in the protein data bank. Proteins 48, 134–140. 10.1002/prot.10150 12012345

[B27] Iakoucheva L. M. Radivojac P. Brown C. J. O'Connor T. R. Sikes J. G. Obradovic Z.. (2004). The importance of intrinsic disorder for protein phosphorylation. Nucleic Acids Res. 32, 1037–1049. 10.1093/nar/gkh253 14960716 PMC373391

[B28] Jacob C. Lebrun-Julien F. Suter U. (2011). How histone deacetylases control myelination. Mol. Neurobiol. 44, 303–312. 10.1007/s12035-011-8198-9 21861092

[B29] Jeong S. G. Cho G. W. (2017). The tubulin deacetylase sirtuin-2 regulates neuronal differentiation through the ERK/CREB signaling pathway, Biochem. Biophys. Res. Commun. 482, 182–187. 10.1016/j.bbrc.2016.11.031 27838300

[B30] Kamat J. P. Devasagayam T. P. (2000). Oxidative damage to mitochondria in normal and cancer tissues, its modulation. Toxicology 155, 73–82. 10.1016/S0300-483X(00)00279-1 11154799

[B31] Klausen M. S. Jespersen M. C. Nielsen H. Jensen K. K. Jurtz V. I. Soenderby C. K.. (2019). NetSurfP-2.0: Improved prediction of protein structural features by integrated deep learning. Proteins. 87, 520–527. 10.1002/prot.25674 30785653

[B32] Korn L. Speicher A. M. Schroeter C. B. Gola L. Kaehne T. Engler A.. (2023). Mapt genotype-dependent mitochondrial aberration and ROS production trigger dysfunction and death in cortical neurons of patients with hereditary FTLD. Redox Biol. 59:102597. 10.1016/j.redox.2022.102597 36599286 PMC9817175

[B33] Krämer A. Green J. Pollard J. Tugendreich S. (2014). Causal analysis approaches in ingenuity pathway analysis. Bioinformatics. 30, 523–530. 10.1093/bioinformatics/btt703 24336805 PMC3928520

[B34] Landgrave-Gómez J. Mercado-Gómez O. Guevara-Guzmán R. (2015). Epigenetic mechanisms in neurological and neurodegenerative diseases. Front. Cell. Neurosci. 9:58. 10.3389/fncel.2015.00058 25774124 PMC4343006

[B35] Liu Y. Geng L. Zhang J. Wang J. Zhang Q. Duan D.. (2018). Oligo-porphyran ameliorates neurobehavioral deficits in parkinsonian mice by regulating the PI3K/Akt/Bcl-2 pathway. Mar. Drugs 16:82. 10.3390/md16030082 29509717 PMC5867626

[B36] Lusardi T. A. Lytle N. K. Gebril H. M. Boison D. (2020). Effects of preinjury and postinjury exposure to caffeine in a rat model of traumatic brain injury. J. Caffeine Adenosine Res 10, 12–24. 10.1089/caff.2019.0012 32181443 PMC7071069

[B37] Marchi U. D. Galindo A. N. Thevenet J. Hermant A. Bermont F. Lassueur S.. (2019). Mitochondrial lysine deacetylation promotes energy metabolism and calcium signaling in insulin-secreting cells, FASEB J. 33, 4660–4674. 10.1096/fj.201801424R 30589571

[B38] Masaldan S. Callegari S. Dewson G. (2022). Therapeutic targeting of mitophagy in Parkinson's disease. Biochem. Soc. Trans. 50, 783–797. 10.1042/BST20211107 35311891 PMC9162468

[B39] Min S. W. Cho S. H. Zhou Y. Schroeder S. Haroutunian V. Seeley W. W.. (2010). Acetylation of tau inhibits its degradation and contributes to tauopathy. Neuron. 67, 953–966. 10.1016/j.neuron.2010.08.044 20869593 PMC3035103

[B40] Min S. W. Sohn P. D. Li Y. Devidze N. Johnson J. R. Krogan N. J.. (2018). SIRT1 Deacetylates tau and reduces pathogenic tau spread in a mouse model of tauopathy. J. Neurosci. 38, 3680–3688. 10.1523/JNEUROSCI.2369-17.2018 29540553 PMC5895994

[B41] Molines L. Nusinovici S. Moreau M. Remy M. May-Panloup P. Flamant C.. (2019). Impact of mode of conception on neonatal and neurodevelopmental outcomes in preterm infants. Hum. Reprod. 34, 356–364. 10.1093/humrep/dey345 30496424

[B42] Ogino S. Lochhead P. Chan A. T. Nishihara R. Cho E. Wolpin B. M.. (2013). Molecular pathological epidemiology of epigenetics: emerging integrative science to analyze environment, host, and disease. Mod. Pathol. 26, 465–484. 10.1038/modpathol.2012.214 23307060 PMC3637979

[B43] Ösz B. E. Jîtcă G. Ştefǎnescu R. E. Puşca Ş, A. Tero-Vescan A. Vari C. E.. (2022). Caffeine and its antioxidant properties-it is all about dose and source. Int. J. Mol. Sci 23:3074. 10.3390/ijms232113074 36361861 PMC9654796

[B44] Pereira-Figueiredo D. Nascimento A. A. Cunha-Rodrigues M. C. Brito R. (2022). Calaza KCaffeine C, and its neuroprotective role in ischemic events: a mechanism dependent on adenosine receptors. Cell. Mol. Neurobiol. 42, 1693–1725. 10.1007/s10571-021-01077-4 33730305 PMC11421760

[B45] Potter M. Rosenkrantz T. Fitch R. (2018). Behavioral and neuroanatomical outcomes in a rat model of preterm hypoxic-ischemic brain Injury: effects of caffeine and hypothermia. Int. J. Dev. Neurosci. 70, 46–55. 10.1016/j.ijdevneu.2018.02.001 29476789

[B46] Pradeepkiran J. A. Baig J. Selman A. Reddy P. H. (2023). Mitochondria in aging and Alzheimer's disease: focus on mitophagy. Neuroscientist 3:10738584221139761. 10.1177/10738584221139761 36597577

[B47] Ruiz-Gabarre D. Carnero-Espejo A. Ávila J. García-Escudero V. (2022). What's in a Gene? The outstanding diversity of Mapt. Cells. 11 10.3390/cells11050840 35269461 PMC8909800

[B48] Samadi M. Shaki F. Bameri B. Fallah M. Ahangar N. Mohammadi H.. (2021). Caffeine attenuates seizure and brain mitochondrial disruption induced by Tramadol: the role of adenosinergic pathway. Drug Chem. Toxicol. 44, 613–619. 10.1080/01480545.2019.1643874 31368376

[B49] Sarnat H. B. Flores-Sarnat L. (2015). Infantile tauopathies: hemimegalencephaly; tuberous sclerosis complex; focal cortical dysplasia 2; ganglioglioma. Brain Dev. 37, 553–562. 10.1016/j.braindev.2014.08.010 25451314

[B50] Shen Y. Wang X. Nan N. Fu X. Zeng R. Yang Y.. (2023). SIRT3-Mediated deacetylation of SDHA rescues mitochondrial bioenergetics contributing to neuroprotection in rotenone-induced PD models. Mol. Neurobiol. 10.1007/s12035-023-03830-w 38087172

[B51] Shi Y.-H. Zhang X.-L. Ying P.-J. Wu Z.-Q. Lin L.-L. Chen W.. (2021). Neuroprotective effect of astragaloside iv on cerebral ischemia/reperfusion injury rats through Sirt1/Mapt pathway. Front. Pharmacol. 12, 639898. 10.3389/fphar.2021.639898 33841157 PMC8033022

[B52] Shu L. Xu C. Q. Yan Z. Y. Yan Y. Jiang S. Z. Wang Y. R.. (2019). Post-stroke microglia induce Sirtuin2 expression to suppress the anti-inflammatory function of infiltrating regulatory T cells. Inflammation. 42, 1968–1979. 10.1007/s10753-019-01057-3 31297748

[B53] Shukla S. Shankavaram U. T. Nguyen P. Stanley B. A. Smart D. K. (2015). Radiation-induced alteration of the brain proteome: understanding the role of the sirtuin 2 deacetylase in a murine model. J. Proteome Res. 14, 4104–4117. 10.1021/acs.jproteome.5b00083 26373435 PMC5028131

[B54] Silva D. F. Esteves A. R. Oliveira C. R. Cardoso S. M. (2017). Mitochondrial metabolism power SIRT2-dependent deficient traffic causing alzheimer's-disease related pathology. Mol. Neurobiol. 54, 4021–4040. 10.1007/s12035-016-9951-x 27311773

[B55] Soustiel J. F. Larisch S. (2010). Mitochondrial damage: a target for new therapeutic horizons. Neurotherapeutics 7, 13–21. 10.1016/j.nurt.2009.11.001 20129493 PMC5084108

[B56] Stilling R. M. Dinan T. G. Cryan J. F. (2014). Microbial genes, brain and behaviour - epigenetic regulation of the gut-brain axis. Genes Brain Behav. 13, 69–86. 10.1111/gbb.12109 24286462

[B57] Tiwari V. Mishra A. Singh S. Shukla S. (2023). Caffeine improves memory and cognition via modulating neural progenitor cell survival and decreasing oxidative stress in Alzheimer's rat model. Curr. Alzheimer Res. 20, 175–189. 10.2174/1567205020666230605113856 37282567

[B58] Toker L. Tran G. T. Sundaresan J. Tysnes O.-B. Alves G. Haugarvoll K.. (2021). Genome-wide histone acetylation analysis reveals altered transcriptional regulation in the Parkinson's disease brain. Mol. Neurodegener. 16:31. 10.1186/s13024-021-00450-7 33947435 PMC8097820

[B59] Trzeciakiewicz H. Tseng J.-H. Wander C. M. Madden V. Tripathy A. Yuan C.-X.. (2017). A dual pathogenic mechanism links tau acetylation to sporadic tauopathy. Sci. Rep. 7, 44102. 10.1038/srep44102 28287136 PMC5347034

[B60] Vannucci R. C. Connor J. R. Mauger D. T.. (1999). Rat model of perinatal hypoxic-ischemic brain damage. J. Neurosci. Res. 55, 158–163. 10.1002/(SICI)1097-4547(19990115)55:2<158::AID-JNR3>3.0.CO;2-1 9972818

[B61] Vongsfak J. Pratchayasakul W. Apaijai N. Vaniyapong T. Chattipakorn N. Chattipakorn S. C.. (2021). The alterations in mitochondrial dynamics following cerebral ischemia/reperfusion injury. Antioxidants (Basel) 10:1384. 10.3390/antiox10091384 34573016 PMC8468543

[B62] Wang B. Zhang Y. Cao W. Wei X. Chen J. Ying W.. (2016). SIRT2 plays significant roles in lipopolysaccharides-induced neuroinflammation and brain injury in mice. Neurochem. Res. 41, 2490–2500. 10.1007/s11064-016-1981-2 27350577

[B63] Wang L.-T. He P.-C. Li A.-Q. Cao K.-X. Yan J.-W. Guo S.. (2021). Caffeine promotes angiogenesis through modulating endothelial mitochondrial dynamics. Acta Pharmacol. Sin. 42, 2033–2045. 10.1038/s41401-021-00623-6 33664417 PMC8632980

[B64] Yang L. Yu X. Zhang Y. Liu N. Li D. Xue X.. (2022a). Proteomic analysis of the effects of caffeine in a neonatal rat model of hypoxic-ischemic white matter damage. CNS Neurosci. Ther. 28, 1019–1032. 10.1111/cns.13834 35393758 PMC9160447

[B65] Yang L. Yu X. Zhang Y. Liu N. Xue X. Fu J.. (2022b). Caffeine treatment started before injury reduces hypoxic-ischemic white-matter damage in neonatal rats by regulating phenotypic microglia polarization, Pediatr. Res. 92, 1543–1554. 10.1038/s41390-021-01924-6 35220399 PMC9771815

[B66] Yang Q. Zhou Y. Sun Y. Luo Y. Shen Y. Shao A.. (2020). Will sirtuins be promising therapeutic targets for TBI and associated neurodegenerative diseases. Front. Neurosci. 14:791. 10.3389/fnins.2020.00791 32848564 PMC7411228

[B67] Yang W. Li X. Jiang G. Long Y. Li H. Yu S.. (2022). Crotonylation versus acetylation in petunia corollas with reduced acetyl-CoA due to PaACL silencing. Physiol. Plant 174. *E*13794. 10.1111/ppl.13794 36193016

[B68] Yang X. J. Seto E. (2007). HATs and HDACs: from structure, function and regulation to novel strategies for therapy and prevention. Oncogene 26, 5310–5318. 10.1038/sj.onc.1210599 17694074

[B69] Younge N. Goldstein R. F. Bann C. M. Hintz S. R. Patel R. M. Smith P. B.. (2017). Survival and neurodevelopmental outcomes among periviable infants. N. Engl. J. Med. 376, 617–628. 10.1056/NEJMoa1605566 28199816 PMC5456289

[B70] Zhang C. C. Xing A. Tan M. S. Tan L. Yu J. T. (2016). The role of mapt in neurodegenerative diseases: genetics, mechanisms and therapy. Mol. Neurobiol. 53, 4893–4904. 10.1007/s12035-015-9415-8 26363795

[B71] Zhao S. Xu W. Jiang W.. (2010). Regulation of cellular metabolism by protein lysine acetylation. Science 327, 1000–1004. 10.1126/science.1179689 20167786 PMC3232675

[B72] Zhu M. Liu M. Guo Q. L. Zhu C. Q. Guo J. C. Prolonged D. A. D. L. E.. (2018). exposure epigenetically promotes Bcl-2 expression and elicits neuroprotection in primary rat cortical neurons via the PI3K/Akt/NF-κB pathway. *Acta Pharmacol*. Sin. 39, 1582–1589. 10.1038/aps.2018.7 29795362 PMC6289365

